# Anti-inflammatory activity of electron-deficient organometallics

**DOI:** 10.1098/rsos.170786

**Published:** 2017-11-29

**Authors:** Jingwen Zhang, Anaïs Pitto-Barry, Lijun Shang, Nicolas P. E. Barry

**Affiliations:** School of Chemistry and Biosciences, University of Bradford, Bradford BD7 1DP, UK

**Keywords:** half-sandwich complexes, carborane, electron-deficient, anti-inflammatory, nitric oxide (NO), mrc-5 fibroblast

## Abstract

We report an evaluation of the cytotoxicity of a series of electron-deficient (16-electron) half-sandwich precious metal complexes of ruthenium, osmium and iridium ([Os/Ru(*η*^6^-*p*-cymene)(1,2-dicarba-*closo*-dodecarborane-1,2-dithiolato)] (**1/2**), [Ir(*η*^5^-pentamethylcyclopentadiene)(1,2-dicarba-*closo*-dodecarborane-1,2-dithiolato)] (**3**), [Os/Ru(*η*^6^-*p*-cymene)(benzene-1,2-dithiolato)] (**4/5**) and [Ir(*η*^5^-pentamethylcyclopentadiene)(benzene-1,2-dithiolato)] (**6**)) towards RAW 264.7 murine macrophages and MRC-5 fibroblast cells. Complexes **3** and **6** were found to be non-cytotoxic. The anti-inflammatory activity of **1–6** was evaluated in both cell lines after nitric oxide (NO) production and inflammation response induced by bacterial endotoxin lipopolysaccharide (LPS) as the stimulus. All metal complexes were shown to exhibit dose-dependent inhibitory effects on LPS-induced NO production on both cell lines. Remarkably, the two iridium complexes **3** and **6** trigger a full anti-inflammatory response against LPS-induced NO production, which opens up new avenues for the development of non-cytotoxic anti-inflammatory drug candidates with distinct structures and solution chemistry from that of organic drugs, and as such with potential novel mechanisms of action.

## 1. Introduction

Inflammation is the body's natural protective response to tissue injury caused by physical or chemical stimuli or invading microbial toxins. Prolonged inflammation can lead to chronic inflammatory diseases such as asthma, rheumatoid arthritis, multiple sclerosis, Parkinson's and Alzheimer's diseases and cancer [1–6]. Pro-inflammatory cells, mainly activated macrophages, produce inflammatory cytokines and other inflammatory mediators, including overproduction of nitric oxide (NO) during the process of inflammation [7]. Inflammation can be initiated and triggered by the stimulation of lipopolysaccharide (LPS), tumour necrosis factor-alpha (TNF-α), interleukin-1 (IL-1) or interferon-gamma (IFN-γ) [8,9]. After exposure to stimulants, the production of inflammatory cytokines and NO are increased in active macrophages [10]. Therefore, inhibition of NO production is a major target for anti-inflammatory drug discovery. Nonetheless, there are severe side-effects associated to current anti-inflammatory drugs and it is of the utmost importance to develop new anti-inflammatory compounds with less side-effects and different mechanisms of action.

Metal coordination complexes offer a versatile platform for the design of drugs with potential unique mechanisms of action. The large pool of metal-based drugs arises from the variety of metals, oxidation states and overall coordination geometry that can be used for their design [11–24]. The number and types of ligands have a strong influence on the activity of the synthesized metal complex. Although very ancient, the utilization of metal ions and metal complexes in medicine has been quite recently stimulated by the clinical success of cisplatin and other platinum(II) anticancer drugs, and by the utilization of gadolinium complexes as MRI contrast agents and 99 m-technetium radiopharmaceuticals for γ-ray imaging. The large structural diversity of metal complexes is also an attractive platform for drug design for other diseases and conditions, including neurodegeneration, microbial and parasitic infections and inflammation [25–28]. We recently surveyed the significant number of clinical trials that involve metal compounds, both for therapy and for diagnosis [29]. In this context, it is clear that metal complexes have the potential to offer an alternative to anti-inflammatory organic drugs. The gold(I) complex auranofin was approved by the FDA in 1985 as an oral anti-arthritic agent. Although still not fully elucidated, [30] its mechanism of action is believed to involve the inhibition of cathepsin B [31]. Bismuth complexes are effective towards a number of diseases, such as syphilis, but the main use of bismuth in therapy is for the inhibition of *Helicobacter pylori* [32,33]. *Helicobacter pylori* is a bacterium that can prevent ulcers from healing [34]. Owing to the development of antibiotic drug resistance by *H. pylori*, triple and quadruple [35] regimens based on two antibiotics, an acid-suppressing agent, and bismuth salts are now being investigated for the treatment of this bacterium.

**Figure 1.**
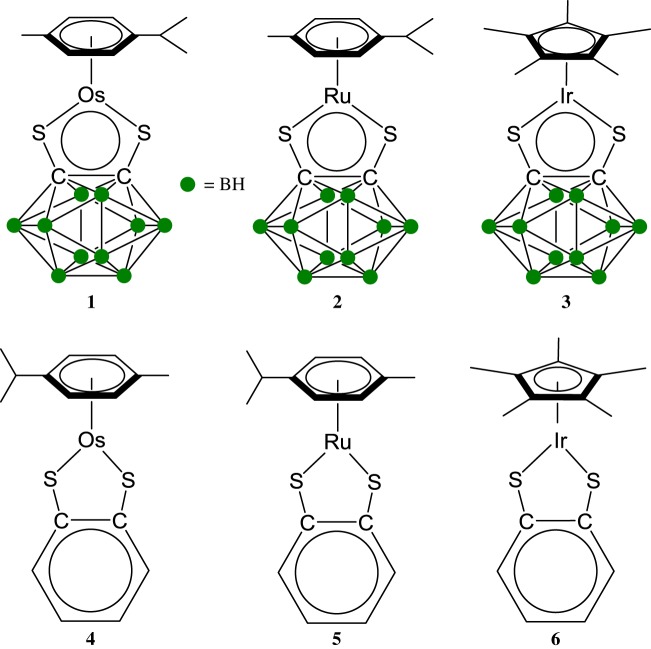
Molecular structures of the electron-deficient half-sandwich metal complexes studied in this work.

Half-sandwich metal complexes of ruthenium, osmium and iridium are a versatile family of organometallics [36–38]. Their biological properties have raised considerable expectations for the treatment of cancer since the beginning of the 2000s and are considered as a promising alternative to platinum-based chemotherapeutics [39–41]. We have recently developed a strong interest in the chemical and biological properties of electron-deficient half-sandwich complexes. In particular, the 16-electron (16-e) complexes based on the carborane ligand [Os(*η*^6^-*p*-cymene)(1,2-dicarba-*closo*-dodecarborane-1,2-dithiolato)] (**1**), [Ru(*η*^6^-*p*-cymene)(1,2-dicarba-*closo*-dodecarborane-1,2-dithiolato)] (**2;** figure 1) have been shown to be highly active towards A2780 ovarian cancer cells [42,43]. Their benzene-dithiolato analogues [Os(*η*^6^-*p*-cymene)(benzene-1,2-dithiolato)] (**4**), [Ru(*η*^6^-*p*-cymene)(benzene-1,2-dithiolato)] (**5**, figure 1) have been recently been shown to exhibit very low reactivity with σ-donor ligands in solution [44]. The lack of reactivity towards pyridine derivatives in particular was found to be related to the aromaticity of the five-membered MS_2_C_2_ chelate ring by involving sulfur lone pairs in the bonding in the MS_2_C_2_ chelate ring.

Here we report an evaluation of the cytotoxicity of electron-deficient metal complexes **1** and **2**, their iridium analogue [Ir(*η*^5^-pentamethylcyclopentadiene)(1,2-dicarba-*closo*-dodecarborane-1,2-dithiolato)] (**3** [45]), and the benzene-dithiol analogues **4**, **5** and [Ir(*η*^5^-pentamethylcyclopentadiene)(benzene-1,2-dithiolato)] (**6** [44] figure 1) towards RAW 264.7 murine macrophages and MRC-5 fibroblast cells, two cell lines widely used as *in vitro* models for the studies of inflammation. The anti-inflammatory activity of **1–6** was evaluated for both cell lines after inflammation response triggered by bacterial endotoxin lipopolysaccharide as the stimulus.

## 2. Material and methods

### 2.1. Materials

#### 2.1.1. Metal complexes

Metals chloride hydrates were purchased from Precious Metals Online. All other reagents were obtained from commercial suppliers and used as received. THF was distilled over calcium hydride. Procedures were performed under nitrogen atmosphere and dried glassware, unless otherwise stated. The [(*η*^6^*-p*-cymene)MCl(μ-Cl)]_2_ (M: Ru and Os) and [(*η*^5^-C_5_(CH_3_)_5_)IrCl(μ-Cl)]_2_ dimers were prepared according to literature procedures [46]. [(*η*^5^-C_5_(CH_3_)_5_)Ir(S_2_C_6_H_4_)] was prepared according to literature procedures [47]. Complexes [Os(*η*^6^-*p*-cymene)(1,2-dicarba-*closo*-dodecarborane-1,2-dithiolato)] (**1**), [Ru(*η*^6^-*p*-cymene)(1,2-dicarba-*closo*-dodecarborane-1,2-dithiolato)] (**2**), [Ir(*η*^5^-pentamethylcyclopentadiene) (1,2-dicarba-*closo*-dodecarborane-1,2-dithiolato)] (**3**)**,** [Os(*η*^6^-*p*-cymene)(1,2-benzene-1,2-dithiolato)] (**4**), [Ru(*η*^6^-*p*-cymene)(1,2-benzene-1,2-dithiolato)] (**5**) and [Ir(*η*^5^-pentamethylcyclopentadiene)(1,2-benzene-1,2-dithiolato)] (**6**) were prepared according to literature procedures [47–49].

#### 2.1.2. Reagents

Dulbecco's Modified Eagle's Medium (DMEM), fetal bovine serum (FBS), penicillin and streptomycin, phosphate-buffered saline (PBS, pH 7.4) and other tissue culture reagents were purchased from Gibco (Thermo Fisher Scientific, UK). LPS, 3-(4,5-dimethyl-2-thiazolyl)-2,5-diphenyltetrazolium bromide (MTT) and Griess reagent were purchased from Promega, and all other chemicals were purchased from Sigma-Aldrich (UK).

#### 2.1.3. Cell culture

RAW 264.7 mouse macrophage cells were obtained from Sigma-Aldrich (UK), and MRC-5 fibroblast cells were provided by Dr Steve Shnyder, University of Bradford. Cells were cultured in DMEM supplemented with 10% FBS containing 100 U ml^–1^ of penicillin and 100 µg ml^–1^ of streptomycin at 37°C in a 5% CO_2_ humidified incubator. The solvent used for all biological experiments was 5 : 95 (v : v) DMSO : water.

#### 2.1.4. Cell viability

Cell viability was assessed using the MTT assay according to the manual. Briefly, cells (2 × 10^4^ cells/well) were seeded in a 96-well plate and treated with compounds. Following treatment, 20 µl of an MTT solution (5 mg ml^−1^ in phosphate-buffered saline) was added to each well and further incubated for 4 h at 37°C. Subsequently, 200 µl of dimethyl sulfoxide (DMSO) was added to each well to solubilize any deposited formazon. The optical density (OD) of each well was measured at 490 nm with a microplate reader (Dynex Technologies).

#### 2.1.5. Quantification of NO production

NO concentration in the cultured medium was determined *via* the Griess reaction. Specifically, 100 µl of supernatant from each well was mixed with 100 µl of Griess reagent (1% sulfanilamide in 5% phosphoric acid and 0.1% naphthylethylenediamine dihydrochloride in water) in a separate 96-well plate. After an incubation of 15 min at ambient temperature, the optical density was determined at 540 nm with a microplate reader.

#### 2.1.6. Statistical analysis

All results are from at least three independent experiments and presented as the mean ± s.d (standard deviation). Significant differences were examined using an analysis of variance (ANOVA) and a Student's *t*-test with SPSS v. 11.0 software (SPSS Inc., Chicago, IL, USA).

#### 2.1.7. Instrumentation

Atmospheric solids analysis probe mass spectroscopy (ASAP-MS) experiments were performed on a Micromass ZMD mass spectrometer. Mass analysis was performed in positive ionization mode. Settings are the following: source temperature 400°C, sampling cone 14 V, corona 3.94 kV.

UV–visible spectroscopy was carried out on a PerkinElmer Lambda 35 UV/visible spectrometer or an Agilent Cary 60 UV–visible spectrophotometer. Quartz cells with two polished sides were used.

## 3. Results

### 3.1. Evaluation of the cytotoxicity of complexes **1–6**

**Figure 2.**
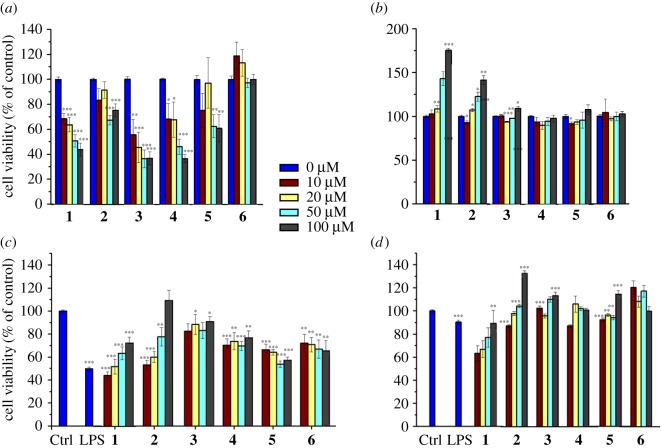
Effects of complexes **1–6** on the viability of RAW 264.7 (*a,b*) and LPS-treated RAW 264.7 (*c,d*) macrophage cells evaluated by the MTT assay; 24 h (*a,c*) after treatment by the complexes and with a further 24 h of recovery time (*b,d*). Detailed data are included in the electronic supplementary material. All statistical analyses refer to the untreated control. *p < 0.05, **p < 0.01, ***p < 0.001.

The cellular viability of RAW 264.7 macrophage cells in the presence of the complexes **1–6** was first investigated (figure 2*a,b*). All complexes show slightly reduced cell viability after 24 h of compounds treatment except complex **6**, which did not induce any significant changes in cell viabilities for all tested concentrations. The cytotoxic effects of complexes **1–5** were found to be dose-dependent (figure 2*a*). Interestingly, no cytotoxicity was observed for all compounds after 48 h including 24 h of recovery, and this was tested for all chosen concentrations (figure 2*b*).

The cytotoxicity of complexes **1–6** towards RAW 264.7 cells in the presence of LPS (1 µg ml^−1^) after 24 h (without recovery time; figure 2*c*) and after 48 h (with 24 h of recovery time; figure 2*d*) was then investigated *via* the MTT assay. After 24 h of treatment by the complexes in the presence of LPS, it was found that LPS significantly reduces the viability of RAW 264.7 cells (Ctrl: 100.00 ± 0.98 versus LPS: 49.77 ± 1.67). Complexes **1–6** also showed reduced cell viability with a slightly varied response among them compared to the LPS group (figure 2*a,c*). Complexes **1** and **2** exhibit a concentration-dependent inhibition, while the other complexes are concentration-independent. Interestingly, when the cells are allowed a 24 h recovery, the compounds do not exhibit any cytotoxicity towards RAW 264.7 cells (with the exception of the slight weak recovery observed with complex **1**; figure 2*c,d*).

**Figure 3.**
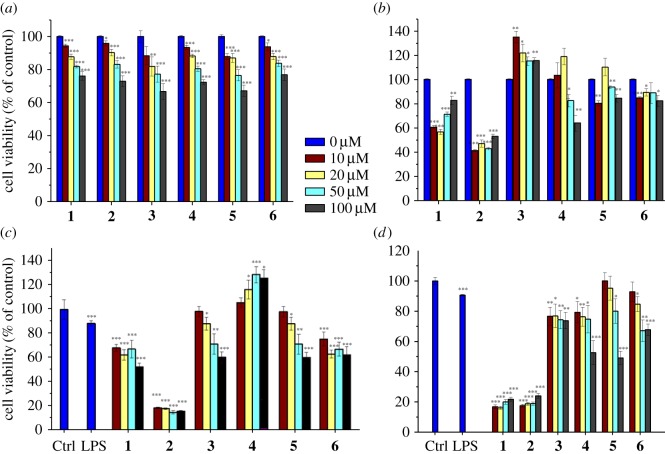
Effects of complexes **1–6** on the viability of MRC-5 fibroblasts (*a,b*) and LPS-treated MRC-5 fibroblast cells (*c,d*) evaluated by the MTT assay; 24 h (*a,c*) after treatment by the complexes and with a further 24 h of recovery time (*b,d*). Detailed data are included in the electronic supplementary material. All statistical analyses refer to the untreated control. *p < 0.05, **p < 0.01, ***p < 0.001.

The MRC-5 fibroblast cell line was then used to investigate if the effect of complexes **1–6** on cell viability is cell type-specific. We tested the cell viability after 24 h of incubation with complexes **1–6**, without recovery time (figure 3*a*) and with a 24 h period of recovery (figure 3*b*). As previously reported [42], complex **2** was found to be highly cytotoxic towards MRC-5 cells, and a strong dose-independent cytotoxic effect was observed, even after 24 h of recovery (figure 3*b*). Interestingly, the other complexes do not show a strong cytotoxic effect. In particular, the two osmium complexes **1** and **4** showed a slight dose-dependent cytotoxicity, while the other complexes have no significant effects on cell viabilities after a 24 h period of recovery. The exposure of the MRC-5 fibroblast cells to LPS (1 µg ml^−1^) after 24 h of incubation (without recovery time; figure 3*c*) and after 48 h (with 24 h of recovery time; figure 3*d*) only slightly induced a reduction in cell viability (Ctrl: 100.00 ± 0.98 versus LPS: 84.97 ± 0.97, *n* = 12). Complexes **1** and **2** were found to be highly cytotoxic with a weak dose-dependent cytotoxicity, with and without recovery time. Complexes **3–6** showed no significant cytotoxicity towards MRC-5 fibroblast cells in the presence of LPS at both 24 and 48 h (see Discussion).

### 3.2. Evaluation of the anti-inflammatory activity of complexes **1–6**

**Figure 4.**
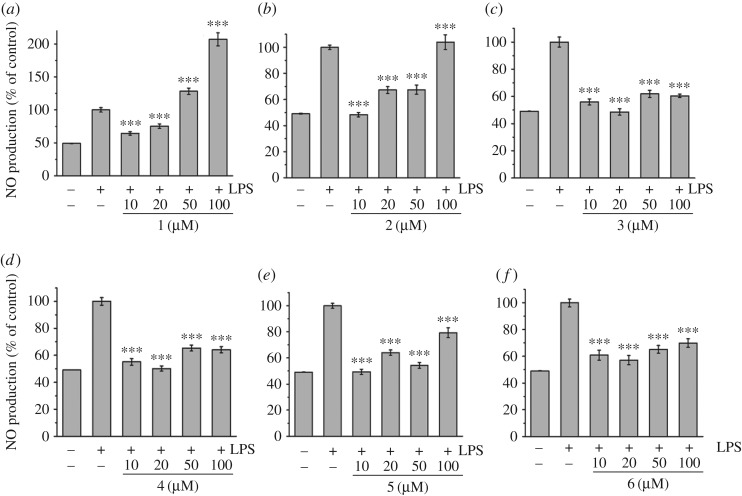
Effects of complexes **1–6** on NO production in LPS-induced RAW 264.7 cells. NO production was measured by the Griess reaction assay and expressed as a percentage of the positive control (LPS alone ‘+ −’). The negative control ‘−−’ looks at the NO production for cells untreated with LPS or any complex. Values are the mean + s.d. of at least three independent experiments. Detailed data are included in the electronic supplementary material. All statistical analyses refer to the positive control (LPS alone ‘+ −’). *p < 0.05, **p < 0.01, ***p < 0.001.

Nitric oxide (NO) production induced by LPS can reflect the degree of inflammation, and a change in the NO level provides a means to assess the effect of agents on the inflammatory process. To determine the effects of complexes **1–6** on the LPS-induced production of NO in RAW 264.7 cells, a cell culture medium was harvested, and the production of NO was measured using the Griess reaction (figure 4). LPS-induced NO production was significantly decreased by the presence of all complexes in a dose-dependent manner at all tested concentrations, except for complexes **1** at concentrations of 50 and 100 µM and **2** at a concentration of 100 µM.

**Figure 5.**
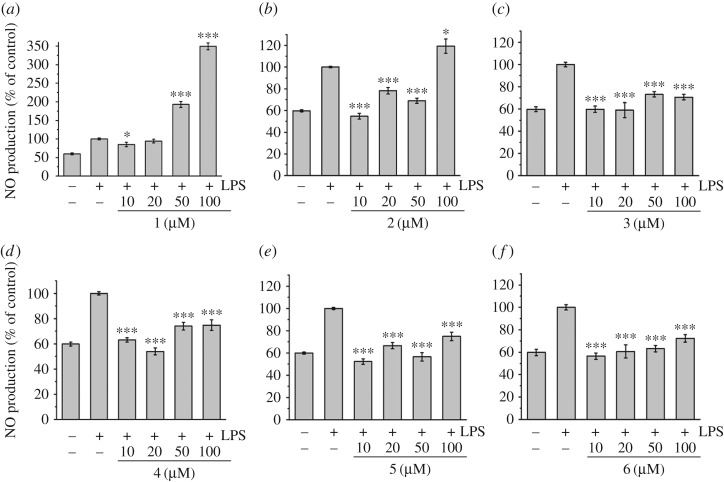
Effects of complexes **1–6** on NO production in LPS-induced MRC-5 fibroblast cells. NO production was measured by the Griess reaction assay and expressed as a percentage of the control (LPS alone ‘+ −’). The negative control ‘− −’ looks at the NO production for cells untreated with LPS or any complex. Values are the mean + s.d. of at least three independent experiments. Detailed data were included in the electronic supplementary material. All statistical analyses refer to the positive control (LPS alone ‘+ −’). *p < 0.05, **p < 0.01, ***p < 0.001.

The LPS-induced production of NO was then measured in LPS-induced MCR-5 cells (figure 5). Similarly, LPS-induced NO production was found to be significantly decreased by the presence of all complexes in a dose-dependent manner at all tested concentrations, except for complexes **1** and **2** at a concentration of 100 µM. Complex **1** was found to exhibit no inhibitor effect on LPS-induced NO production at all tested concentrations. Interestingly, in the case of the iridium complexes **3** and **6,** the decreased NO production reached the level of the control (cells untreated with LPS and complex **‘**− −**’**), which suggests a full anti-inflammation effect for these two compounds.

### 3.3. Cell-free investigation of the formation of NO adducts

**Figure 6.**
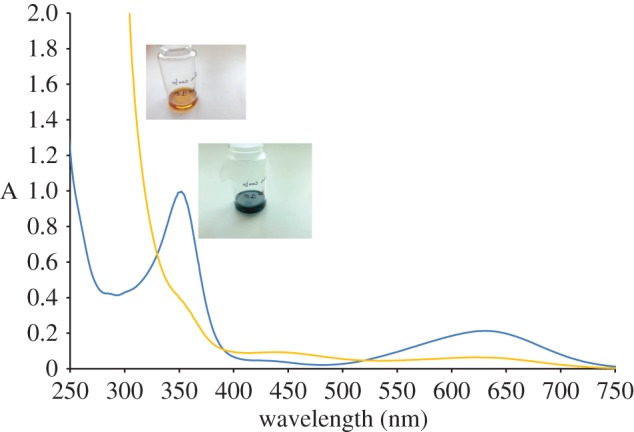
UV–visible spectra of complexes **2** and **[2-NO**] in acetone (10^−4^ M, 298 K), and pictures of the corresponding solutions.

To confirm the ability of the electron-deficient metal complexes **1–6** to interact with nitric oxide, the reactivity of complex **2** with NO in solution was studied. Bubbling NO in an acetone solution (2 × 10^−3^ M, 298 K) of the 16-e complex **2** readily leads to the formation of the 18-e adduct [Ru(*η*^6^-*p*-cymene)(1,2-dicarba-*closo*-dodecarborane-1,2-dithiolato)(NO)] ([**2-NO**]). The addition of the NO ligand is evidenced by a change of the solution colour from blue to yellow (figure 6), which is in accordance with the formation of 18-e species for complexes **1–6** [48]. UV–visible absorption spectroscopy also suggests the formation of the NO 18-e adduct (figure 6), with the dramatic decrease of the absorption band observed at 632 nm (*ϵ* = 2100 l mol^−1^ cm^−1^; associated to ligand-to-metal charge-transfer transitions that are commonly observed in 16-electron complexes [49]). ESI-MS analysis of the *S,S*-coordinated complexes revealed *m/z* peaks that correspond to [**2-NO**] and to the fragmented 16-electron precursor **2** (electronic supplementary material, figure S1).

## 4. Discussion

Electron-deficient organometallics play a key role as intermediates in organometallic reactions [50]. They are also known to be extremely unstable and most organometallics follow the 18-electron rule. Some stable coordinatively unsaturated 16-electron (16-e) complexes have been isolated in particular by the groups of Koelle, Tilley, Suzuki, among others, [51–61], but little is known about their biological properties. Complexes **1–6** are among the rare examples of stable (in air and in solution) electron-deficient organometallics. Furthermore, such complexes exhibit unusual chemistry: Although remarkably stable as 16-e monomer species (owing to the steric hindrance of the bulky carborane, which prevents the dimerization of the compounds and the formation of more electronically favoured 18-e species), [42] these complexes react with σ-donor and π-acceptor ligands to give 18-electron complexes, such as complexes [Ru/Os(*p*-cym)(1,2-dicarba-*closo*-dodecarborane-1,2-dithiolato)(triphenylphosphine)] [48]. When reacted with σ-donor aromatic amines in dichloromethane and chloroform solutions at ambient temperature, the 16-e blue (Ru) and red (Os) complexes are in equilibrium with their yellow 18-electron adducts, and the thermal displacement of the equilibrium results in marked thermochromic properties [62]. Owing to their unusual reactivity in solution, it is anticipated that these 16-e complexes may possess interesting biological properties, and we recently reported the potent anti-proliferative activity of complexes **1** and **2** towards A2780 ovarian cancer cells [42]. However, the effects of these complexes on pro-inflammatory mediators secreted from macrophages and fibroblasts have not been reported. In the present study, we investigated the cytotoxicity of complexes **1–6** and their anti-inflammatory activity on lipopolysaccharide (LPS)-activated RAW 264.7 macrophages and MRC-5 fibroblast cells.

### 4.1. Structure–activity relationships

#### 4.1.1. Inertness, kinetics and electron deficiency: the role of the metal ions

It is clear that some of the complexes studied show distinctive cytotoxic effects in specific cell lines. In principle, low-spin d^6^ half-sandwich complexes [(arene)M(X)(Y)(Z)] (arene = benzene or cyclopentadienyl derivative, (M = Ru, Os or Ir) can be relatively inert towards substitution reactions and exert their anticancer activity by binding to target sites through outer-sphere interactions [63]. This inertness depends on the specific ligand sets. Inert octahedral metal complexes are emerging as promising scaffolds for targeting kinase active sites, [64] owing to their rigid and globular shapes, and high synthetic versatility [65]. Meggers and co-workers have demonstrated that specific Ru^II^, [66] Os^II^, [67] Rh^III^, [68] and Ir^III^ [69] complexes can serve as highly potent (micromolar to nanomolar range) and selective inhibitors of kinases. Osmium, the heavier congener of ruthenium and a third row transition metal, commonly exhibits slower kinetics than ruthenium, and is often considered to be relatively inert. However, it is possible to tune the biochemical reactivity of the arene Os^II^ complexes through an understanding of their aqueous solution chemistry [63]. We previously reported that the Os complexes **1** and **4** are prompt to react with σ-donor ligands [62]. Ruthenium half-sandwich complexes are usually more reactive than their osmium counterparts, and their activation via redox pathways or by ligand substitution can be finely controlled, offering activation only after the complex has reached its target [70].

Although the mechanism of action for the cytotoxicity of complexes **1–6** is unknown and is currently under investigation, it seems clear that the electron deficiency of such 16-e complexes should allow interactions with a number of biomolecules en route to or in cells. In this work, we illustrated such ability by studying the reactivity of complex **2** with NO, which we hypothesize as being the reason for their anti-inflammatory effects. The absence of cytotoxicity for the iridium complexes **3** and **6** may be due to a greater inertness and slower kinetics of Ir than Ru and Os analogues. This is in accordance with our recent study on the reactivity of complexes **4–6** towards σ-donor and σ-donor + π-acceptor ligands [44]. For example, complexes **5** and **6** are only present as the monomeric 16-e species in solution, while the less kinetically inert complex **4** is present as both 16-e monomeric and 18-e dimeric structures in solution. Therefore, the iridium complexes could react with NO (which is an excellent ligand owing to π-back bonding and steric considerations) but not with large biomolecules, while the more kinetically active Ru and Os analogues could react not only with NO but also with other targets (e.g. glutathione) leading to cytotoxicity.

The impact of the nature of the metal ion is evidenced by the fact that the two ruthenium complexes **2** and **5** have a similar effect on the cellular viability of RAW 264.7 macrophage cells at a 20 μM concentration, while the two osmium complexes **1** and **4** exhibit a slight dose-dependent cytotoxicity towards MRC-5 fibroblast cells. The most striking result in terms of structure–activity relationships for the cytotoxicity of the complexes towards the two different cell lines is the observation that the iridium complexes **3** and **6** do not show any cytotoxic effects at any concentrations.

#### 4.1.2. Amphiphilicity: the role of the non-innocent ligands

One of the main reasons for the intensive research on the synthesis of arene-ruthenium based anticancer drug candidates is their amphiphilic properties provided by the hydrophobic arene ligand counterbalanced by the hydrophilic metal centre. Beyond non-classical bonding interactions [71], dicarba-*closo*-dodecarboranes possess unusual properties, including high symmetry, remarkable stability and high hydrophobicity. Dicarba-*closo*-dodecarboranes and dicarba-*closo*-dodecarborane derivatives have been used as pharmacophores to enhance the hydrophobic interactions of pharmaceutical compounds with oestrogen and retinoid receptors [72]. The hydrophobicity of complexes **1–3** is, therefore, expected to be much higher than the one of their benzene analogues. Remarkably, the cytotoxicity results of complexes **1–6** on both cell lines highlight a dramatic difference of activity between the two carborane-containing group VIII metal (Ru and Os) complexes **1** and **2** and the other metal complexes studied in this work. Complexes **1** and **2** show a concentration-dependent inhibition towards RAW 264.7 cells treated with LPS (1 µg ml^−1^), while the activity of the other metal complexes **3–6** was found to be concentration-independent. These same two complexes are the only compounds which exhibit a significant cytotoxicity effect on MRC-5 fibroblast cells treated with LPS. It seems clear that the main factor of activity in the two series of electron-deficient half-sandwich metal complexes, therefore, seems to be the role of the metal ion (because the Ir carborane complex **3** is non-cytotoxic despite incorporating the dicarba-*closo*-dodecarborane non-innocent ligand). The effect of the chelating ligand, although less pronounced than the influence of the nature of the metal ion, is nonetheless of importance, as exemplified by the dramatic difference of behaviour between the carborane- and benzene-containing Os and Ru complexes (**1** versus **4**, and **2** versus **5**). Remarkably, the anti-inflammatory activity of the different complexes follows the same trends of structure–activity relationship as the anti-proliferative one. Carborane- and benzene-containing Ru and Os complexes (**1** versus **4**, and **2** versus **5**) show stark differences in their abilities to decrease the LPS-induced NO production. Finally, it should be noted that all complexes are neutral and have similar solubilities in the mixture 5 : 95 v : v DMSO : water used for the biological testing.

#### 4.1.3. Anti-proliferative activity

Similarly to the cytotoxicity results, the two iridium complexes **3** and **6** display a remarkable difference of behaviour compared to their ruthenium and osmium analogues. Both iridium complexes, in addition to being non-cytotoxic, possess the ability to reduce the NO production to the level of the control assay (cells ‘− −’, untreated with LPS and complex). This indicates that these two compounds are not toxic and offer a full anti-inflammation protection to the cells. Some differences of anti-inflammatory activity are noticeable depending on the cell types, particularly in terms of inhibition of LPS-induced NO production. Although all complexes displayed dose-dependent inhibitory effects on LPS-induced NO production on both cell lines, the compounds were found to have greater potential to inhibit NO production on MCR-5 cells regardless of the metal ions and ligands, than on RAW 264.7 cells, as evidenced by the molar concentration values (μM) giving 50% inhibition (IC_50_) relative to the control in table 1.

**Table 1.** Inhibition of NO production in LPS-induced RAW 264.7 macrophages and MRC-5 fibroblast cells by compounds **1–6**. Inhibition effects are represented as the molar concentration (μM) giving 50% inhibition (IC_50_) relative to the control. IC_50_ values were determined by plotting NO production (percentage of control, *y*-axis) against the complex × concentrations (*x*-axis) from the data in figures 4 and 5. These data represent the average values of three independent experiments.

IC_50_ values (μM)		
complex	MCR-5 cells	RAW 264.7 cells
**1**	>100	12.34 ± 1.19
**2**	4.52 ± 1.95	44.99 ± 4.07
**3**	22.50 ± 9.67	70.34 ± 5.42
**4**	11.41 ± 5.92	57.23 ± 3.36
**5**	24.63 ± 7.50	62.50 ± 4.42
**6**	25.22 ± 3.64	43.27 ± 5.18

## 5. Conclusion

The cytotoxicity and anti-inflammatory effects of six electron-deficient half-sandwich metal complexes of ruthenium, osmium and iridium towards LPS-induced RAW 264.7 macrophages and MRC-5 fibroblast cells were investigated. The six metal complexes were shown to exhibit dose-dependent inhibitory effects on LPS-induced NO production on both cell lines. Remarkably, the two iridium complexes [Ir(*η*^5^-pentamethylcyclopentadiene)(1,2-dicarba-*closo*-dodecarborane-1,2-dithiolato)] (**3**) and [Ir(*η*^5^-pentamethylcyclopentadiene)(benzene-1,2-dithiolato)] (**6**) were found to be non-cytotoxic and to trigger a full anti-inflammatory response towards LPS-induced NO production. We hypothesize that the electron-deficient configuration at the metal centre allows binding of the ligand NO to the metal ion. The extent of such binding and its implication towards the anti-inflammatory mechanisms of action of complexes **1–6** will be investigated in future work. The anti-inflammatory effect of non-cytotoxic 16-e complexes has the potential to be useful in the treatment of severe infectious diseases which trigger inflammation and fibrosis at the end stage, like SARS (severe acute respiratory syndrome), [73] which is a fatal disease without effective antiviral agents available now. Another disease enduring similar pathologic changes is chronic liver infectious disease, like hepatitis C, which also has poor prognosis without proper treatment available. Therefore, the two iridium compounds have the potential to be useful in controlling the progression of these severe diseases; in comparison, cortisone has similar anti-inflammatory effect but causes side effects such as osteonecrosis of the femoral head at high dosage.

## Supplementary Material

Mass spectrometry analysis of a nitric oxide adduct

## Supplementary Material

data sheet

## Supplementary Material

Figures

## Data accessibility

The article's supporting data have been made available as electronic supplementary material.
