# Supplementary material for: Anti-inflammatory activity of electron-deficient organometallics
Source: R Soc Open Sci. 2017 Nov 29;4(11):170786. doi: 10.1098/rsos.170786 (PMC5717645; doi:10.1098/rsos.170786)
Supplement: Mass spectrometry analysis of a nitric oxide adduct [file rsos170786supp1.docx]

Supporting Data for

Anti-inflammatory activity of non-cytotoxic electron-deficient precious metal complexes

Jingwen Zhang, Anaïs Pitto-Barry, Lijun Shang, and Nicolas P. E. Barry

School of Chemistry and Biosciences, University of Bradford, Bradford BD7 1DP, United Kingdom


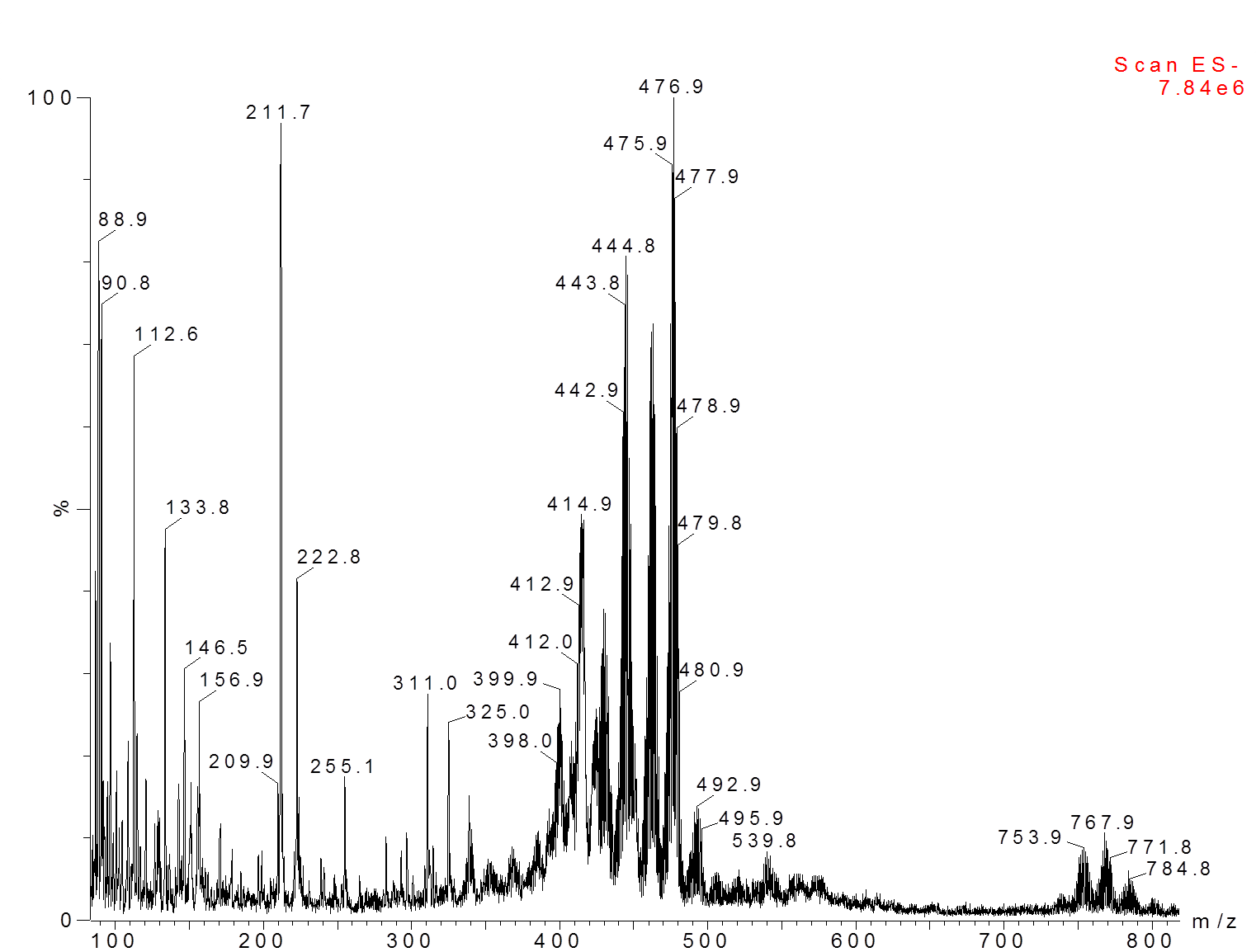


Figure S1. Mass spectrum of complex [**2-NO**] in acetone solution, recorded in ESI- mode.

**MTT assay MRC-5 48hr without LPS (n=3)**

| MRC-5 | 4 | 2 | 3 |
| --- | --- | --- | --- |
| 0uM | 100±0.76 | 100±0.3 | 100±0.29 |
| 10uM | 103.39±10.4 | 41.25±0.84 | 134.96±4.81 |
| 20 | 118.85±6.8 | 46.96±2.99 | 121.79±7.27 |
| 50 | 82.49±5.06 | 42.73±1.18 | 115.22±3.54 |
| 100 | 63.97±6.7 | 53.01±1.82 | 115.55±2.49 |

**MTT assay MRC-5 48 hr with LPS (n=6)**

| MRC-5 | 4 | 2 | 3 |
| --- | --- | --- | --- |
| 10uM | 79.30±7.14 | 17.49±1.01 | 76.69±5.94 |
| 20 | 76.30±6.33 | 18.78±0.99 | 76.89±7.81 |
| 50 | 74.78±9.19 | 18.86±1.07 | 74.37±6.06 |
| 100 | 52.59±8.08 | 24.04±1.6 | 73.76±5.43 |
| MRC-5 | 5 | 6 | 1 |
| 10uM | 100.07±5.41 | 92.85±6.49 | 16.77±1.27 |
| 20 | 95.21±7.93 | 84.68±5.01 | 16.07±0.97 |
| 50 | 80.08±8.09 | 67.10±7.18 | 19.99±1.56 |
| 100 | 49.05±4.42 | 67.77±3.79 | 21.77±1.33 |

| **MRC-5** |  |
| --- | --- |
| Ctrl | 100±2.47 |
| LPS | 90.6±0.48 |

**MTT assay MRC-5 24hr without LPS (n=9)**

| **MRC -5 24hr without LPS** | **0uM** | **10uM** | **20uM** | **50uM** | **100uM** |
| --- | --- | --- | --- | --- | --- |
| 1 | 100±0.67 | 94.35±1.1*** | 87.76±1.65*** | 81.8±0.85*** | 76.11±2.61*** |
| 2 | 100±0.58 | 95.87±1.57* | 90.31±1.99*** | 83.16±2.25*** | 72.9±3.34*** |
| 3 | 100±3.56 | 88.36±5.54 | 81.88±5.81** | 77.26±4.89*** | 66.7±4.78*** |
| 4 | 100±0.76 | 93.4±1.25*** | 88.26±1.1*** | 80.49±1.63*** | 72.33±1.5*** |
| 5 | 100±1.04 | 87.88±1.71*** | 87.03±2.68*** | 76.48±3.37*** | 67.12±3.41*** |
| 6 | 100±0.49 | 93.75±2.41* | 87.89±1.97*** | 83.81±1.94*** | 76.87±3.48*** |

**MTT assay MCR-5 24hr with LPS(n=12)**

| MRC -5 24hr with LPS | 10uM | 20uM | 50uM | 100uM |
| --- | --- | --- | --- | --- |
| 1 | 68.1±2.83*** | 62.28±4.46*** | 67.09±7.45*** | 52.28±3*** |
| 2 | 18.59±0.74*** | 18±0.54*** | 14.92±1.35*** | 15.77±0.77*** |
| 3 | 67.02±3.36 | 64.13±4.33* | 64.5±3.47** | 64.62±3.43*** |
| 4 | 105.82±3.78 | 116.61±7.8* | 129.06±6.76*** | 126.13±6.98* |
| 5 | 98.27±4.3 | 88.26±5.3* | 71.46±8.33** | 60.49±4.3*** |
| 6 | 75.61±5.65*** | 63.15±3.34*** | 67.16±5.81*** | 62.46±6.88*** |

| **MRC-5** |  |
| --- | --- |
| Ctrl | 100±4.14 |
| LPS | 88.56±2.22*** |

**MTT assay RAW 48hr without LPS (n=3)**

| RAW 48hr without LPS | 0uM | 10uM | 20uM | 50uM | 100uM |
| --- | --- | --- | --- | --- | --- |
| 1 | 100±1.45 | 102.68±4.76 | 108.56±3.57** | 142.88±7.9 | 175.31±2.22*** |
| 2 | 100±1.45 | 92.78±1.89* | 107.38±1.67* | 122.57±5.2* | 141.24±5.04** |
| 3 | 100±0.58 | 99.96±2.23 | 93.72±0.49*** | 97.58±0.48** | 108.68±2.49* |
| 4 | 100±1.15 | 93.71±5.11 | 89.69±4.35 | 94.17±4.42 | 97.63±3.89 |
| 5 | 100±2 | 91.63±1.92* | 93.39±2.62 | 95.73±9.02 | 107.62±5.6 |
| 6 | 100±1.76 | 104.01±15.64 | 96.84±2.38 | 99.75±5.03 | 102.26±3.07 |

**MTT assay RAW 48hr with LPS (n=18)**

| RAW 48hr  With LPS | 10uM | 20uM | 50uM | 100uM |
| --- | --- | --- | --- | --- |
| 1 | 63.15±6.8 | 66.77±7.27 | 77.01±8.43 | 89.20±11.22** |
| 2 | 86.96±1.29*** | 97.88±1.85 | 104.32±1.43*** | 132.53±2.19*** |
| 3 | 102.39±1.78 | 95.79±1.69*** | 110.05±2.22 | 113.35±3.11*** |
| 4 | 86.87±1.19 | 105.81±6.92 | 101.87±1.73 | 100.69±1.68 |
| 5 | 92.19±1.53*** | 96.32±1.23** | 94.28±1.92** | 114.52±3.13*** |
| 6 | 120.29±5.77 | 108.20±4.88 | 117.22±5.08 | 99.88±4.16 |

| RAW |  |
| --- | --- |
| Ctrl | 100±1.04 |
| LPS | 90.35±1.7*** |

**MTT assay RAW 24r without LPS (n=9)**

| RAW 24hr without LPS | 0uM | 10uM | 20uM | 50uM | 100uM |
| --- | --- | --- | --- | --- | --- |
| 1 | 100±0.02 | 68.40±4.17*** | 63.74±5.7*** | 50.84±4.89*** | 43.87±5.11*** |
| 2 | 100±0.01 | 83.28±9.28 | 91.43±6.75 | 67.27±3.71*** | 75.24±5.25*** |
| 3 | 100±0.02 | 55.73±12** | 45.38±12.19*** | 36.34±7.25*** | 36.79±5.32*** |
| 4 | 100±0.01 | 68.09±12.48* | 67.67±14.1* | 46.07±6.04*** | 36.44±3.26*** |
| 5 | 100±0.03 | 75.22±13.59 | 97.13±20.25 | 62.43±9.13** | 60.83±10.95** |
| 6 | 100±0.0028 | 118.88±10.97 | 113.29±10.63 | 97.39±3.73 | 100.04±3.94 |

**MTT assay RAW 48hr with LPS (n=18)**

| RAW 48hr  With LPS | 10uM | 20uM | 50uM | 100uM |
| --- | --- | --- | --- | --- |
| 1 | 63.15±6.8 | 66.77±7.27 | 77.01±8.43 | 89.20±11.22** |
| 2 | 86.96±1.29*** | 97.88±1.85 | 104.32±1.43*** | 132.53±2.19*** |
| 3 | 102.39±1.78 | 95.79±1.69*** | 110.05±2.22 | 113.35±3.11*** |
| 4 | 86.87±1.19 | 105.81±6.92 | 101.87±1.73 | 100.69±1.68 |
| 5 | 92.19±1.53*** | 96.32±1.23** | 94.28±1.92** | 114.52±3.13*** |
| 6 | 120.29±5.77 | 108.20±4.88 | 117.22±5.08 | 99.88±4.16 |

| **RAW** |  |
| --- | --- |
| **Ctrl** | **100±1.04** |
| **LPS** | **90.35±1.7***** |

**Effects of compounds 1 – 6 on NO production in LPS-induced MRC-5 cells. (*P<0.05,**P<0.01,***P<0.001)**

| Proinflammatory mediator | | Control (LPS only) | Concentration (μg/mL) of compunds | | | |
| --- | --- | --- | --- | --- | --- | --- |
|  |  |  | 10 | 20 | 50 | 100 |
| **4** | NO after 24 h incubation (%) | 100 ±1.36 | 63.15±1.68*** | 53.94±2.73*** | 74.04±3.02*** | 74.87±4.30*** |
| **2** | NO after 24 h incubation (%) | 100±0.75 | 54.77±2.59*** | 78.22±3.09*** | 69.01±2.40*** | 119.26±6.54* |
| **3** | NO after 24 h incubation (%) | 100±2.05 | 59.80±2.90*** | 58.96±6.67*** | 73.20±2.48*** | 70.69±2.40*** |
| **5** | NO after 24 h incubation (%) | 100±0.97 | 52.26±2.51*** | 66.50±2.80*** | 56.45±3.82*** | 74.87±3.89*** |
| **6** | NO after 24 h incubation (%) | 100±2.41 | 56.45±2.80*** | 60.64±5.86*** | 63.15±2.80*** | 72.36±3.37*** |
| **1** | NO after 24 h incubation (%) | 100±2.51 | 84.92±6.09* | 94.14±4.57 | 192.96±7.76*** | 349.58±9.42*** |

**Effects of compounds 1 – 6 on NO production in LPS-induced RAW264.7 macrophages.**

| Proinflammatory mediator | | Control (LPS only) | Concentration (μg/mL) of compunds | | | |
| --- | --- | --- | --- | --- | --- | --- |
|  |  |  | 10 | 20 | 50 | 100 |
| 4 | NO after 24 h incubation (%) | 100 ±2.72 | 55.15±2.47*** | 50.22±1.95  *** | 65.40±2.23  *** | 64.18±2.37  *** |
| 2 | NO after 24 h incubation (%) | 100±1.78 | 48.38±1.62  *** | 67.25±2.66  *** | 67.52±3.47  *** | 103.98±5.70  *** |
| 3 | NO after 24 h incubation (%) | 100±3.64 | 55.96±2.19  *** | 48.60±2.26  *** | 61.92±2.57  *** | 60.45±1.24  *** |
| 5 | NO after 24 h incubation (%) | 100±1.97 | 49.39±1.96  *** | 64.13±2.00  *** | 54.31±2.25  *** | 79.36±3.73  *** |
| 6 | NO after 24 h incubation (%) | 100±2.92 | 60.80±3.77  *** | 57.10±3.50  *** | 65.14±2.87*** | 69.87±3.22*** |
| 1 | NO after 24 h incubation (%) | 100±3.33 | 64.38±2.62*** | 75.44±3.33*** | 128.21±4.90*** | 206.83±9.93*** |
