# Supplementary material for: Anti-inflammatory activity of electron-deficient organometallics
Source: R Soc Open Sci. 2017 Nov 29;4(11):170786. doi: 10.1098/rsos.170786 (PMC5717645; doi:10.1098/rsos.170786)
Supplement: data sheet [file rsos170786supp2.pdf]

MCR-5 48hr without LPS (n=3)

MCR-5	CE32 4	JT020 2	CE40 3
0uM	100±0.76	100±0.3	100±0.29
10uM	103.39±10.4	41.25±0.84	134.96±4.81
20	118.85±6.8	46.96±2.99	121.79±7.27
50	82.49±5.06	42.73±1.18	115.22±3.54
100	63.97±6.7	53.01±1.82	115.55±2.49

MCR-5	CE44 5	CE47 6	CE49 1
0uM	100±0.23	100±0.58	’100±0.27
10uM	80.25±2.1	84.82±0.76	60.50±1.41
20	110.20±7.22	89.28±3.04	56.65±2.19
50	93.73±1.1	88.91±8.52	71.27±1.86
100	84.46±3.13	82.32±4.44	82.67±3.53

±2.47

±0.48

MCR-5 48 hr with LPS (n=6)

MCR-5	CE32 4	JT020 2	CE40 3
10uM	79. 30±7. 14	17. 49±1. 01	76. 69±5. 94
20	76. 30±6. 33	18. 78±0. 99	76. 89±7. 81
50	74. 78±9. 19	18. 86±1. 07	74. 37±6. 06
100	52. 59±8. 08	24. 04±1. 6	73. 76±5. 43

MCR-5	CE44 5	CE47 6	CE49 1
10uM	100. 07±5. 41	92. 85±6. 49	16. 77±1. 27
20	95. 21±7. 93	84. 68±5. 01	16. 07±0. 97
50	80. 08±8. 09	67. 10±7. 18	19. 99±1. 56
100	49. 05±4. 42	67. 77±3. 79	21. 77±1. 33

MCR-5 24hr without LPS (n=9)

MCR -5 24hr without LPS	0uM	10uM	20uM	50uM	100uM
1	100±0.67	94.35±1.1***	87.76±1.65***	81.8±0.85***	76.11±2.61***
2	100±0.58	95.87±1.57*	90.31±1.99***	83.16±2.25***	72.9±3.34***
3	100±3.56	88.36±5.54	81.88±5.81**	77.26±4.89***	66.7±4.78***
4	100±0.76	93.4±1.25***	88.26±1.1***	80.49±1.63***	72.33±1.5***
5	100±1.04	87.88±1.71***	87.03±2.68***	76.48±3.37***	67.12±3.41***
6	100±0.49	93.75±2.41*	87.89±1.97***	83.81±1.94***	76.87±3.48***

MCR-5	
Ctrl	100±4.14
LPS	88. 56±2. 22***

MCR-5 24hr with LPS(n=12)

MCR -5 24hr with LPS	10uM	20uM	50uM	100uM
1	68. 1±2. 83***	62. 28±4. 46***	67. 09±7. 45***	52. 28±3***
2	18. 59±0. 74***	18±0. 54***	14. 92±1. 35***	15. 77±0. 77***
3	67. 02±3. 36	64. 13±4. 33*	64. 5±3. 47**	64. 62±3. 43***
4	105. 82±3. 78	116. 61±7. 8*	129. 06±6. 76***	126. 13±6. 98*
5	98. 27±4. 3	88. 26±5. 3*	71. 46±8. 33**	60. 49±4. 3***
6	75. 61±5. 65***	63. 15±3. 34***	67. 16±5. 81***	62. 46±6. 88***

Table 2-1. Effects of compounds 1-6 on nitrite (NO) production in LPS-induced MCR-5 macrophages.
(*P<0.05,**P<0.01,***P<0.001)

Proinflammatory mediator		Control (LPS only)	Concentration (μg/mL) of compunds			
			10	20	50	100
CE32	NO after 24 h incubation (%)	100 ±1.36	63.15±1.68***	53.94±2.73***	74.04±3.02***	74.87±4.30***
JT020	NO after 24 h incubation (%)	100±0.75	54.77±2.59***	78.22±3.09***	69.01±2.40***	119.26±6.54*
CE40	NO after 24 h incubation (%)	100±2.05	59.80±2.90***	58.96±6.67***	73.20±2.48***	70.69±2.40***
CE44	NO after 24 h incubation (%)	100±0.97	52.26±2.51***	66.50±2.80***	56.45±3.82***	74.87±3.89***
CE47	NO after 24 h incubation (%)	100±2.41	56.45±2.80***	60.64±5.86***	63.15±2.80***	72.36±3.37***
CE49	NO after 24 h incubation (%)	100±2.51	84.92±6.09*	94.14±4.57	192.96±7.76***	349.58±9.42***

Table 2-2. Effects of compounds 1-6 on nitrite (NO) production in LPS-induced RAW264.7 macrophages.

Proinflammatory mediator		Control (LPS only)	Concentration (μg/mL) of compunds			
			10	20	50	100
CE32	NO after 24 h incubation (%)	100 ±2.72	55.15±2.47***	50.22±1.95 ***	65.40±2.23 ***	64.18±2.37 ***
JT020	NO after 24 h incubation (%)	100±1.78	48.38±1.62 ***	67.25±2.66 ***	67.52±3.47 ***	103.98±5.70 ***
CE40	NO after 24 h incubation (%)	100±3.64	55.96±2.19 ***	48.60±2.26 ***	61.92±2.57 ***	60.45±1.24 ***
CE44	NO after 24 h incubation (%)	100±1.97	49.39±1.96 ***	64.13±2.00 ***	54.31±2.25 ***	79.36±3.73 ***
CE47	NO after 24 h incubation (%)	100±2.92	60.80±3.77 ***	57.10±3.50 ***	65.14±2.87 ***	69.87±3.22 ***
CE49	NO after 24 h incubation (%)	100±3.33	64.38±2.62***	75.44±3.33 ***	128.21±4.90***	206.83±9.93***

RAW	
Ctrl	100±0.98
LPS	49. 77±1. 67***

RAW 24hr with LPS (n=12)

RAW 24hr with LPS	10uM	20uM	50uM	100uM
1	44. 65±3. 17***	52. 29±6. 48***	64. 18±5. 51***	73. 16±5. 22***
2	53. 25±4***	59. 97±4. 68***	77. 66±8. 01**	109. 08±8. 84
3	82. 41±6. 52	88. 31±8. 63*	83. 05±6. 97	90. 71±4. 52*
4	70. 33±5. 45***	73. 77±7. 46**	69. 67±4. 05***	76. 93±5. 97**
5	67. 18±5. 01***	64. 88±2. 48***	54. 35±2. 82***	57. 81±2. 93***
6	72. 65±7. 84**	71. 27±6. 31**	67. 36±8. 09**	65. 81±8. 88**

RAW	
Ctrl	100±1.04
LPS	90.35±1.7***

RAW 48hr with LPS (n=18)

RAW 48hr With LPS	10uM	20uM	50uM	100uM
1	63. 15±6. 8	66. 77±7. 27	77. 01±8. 43	89. 20±11. 22**
2	86. 96±1. 29***	97. 88±1. 85	104. 32±1. 43***	132. 53±2. 19***
3	102. 39±1. 78	95. 79±1. 69***	110. 05±2. 22	113. 35±3. 11***
4	86. 87±1. 19	105. 81±6. 92	101. 87±1. 73	100. 69±1. 68
5	92. 19±1. 53***	96. 32±1. 23**	94. 28±1. 92**	114. 52±3. 13***
6	120. 29±5. 77	108. 20±4. 88	117. 22±5. 08	99. 88±4. 16

RAW 24r without LPS (n=9)

RAW 24hr without LPS	0uM	10uM	20uM	50uM	100uM
1	100±0.02	68.40±4.17***	63.74±5.7***	50.84±4.89***	43.87±5.11***
2	100±0.01	83.28±9.28	91.43±6.75	67.27±3.71***	75.24±5.25***
3	100±0.02	55.73±12**	45.38±12.19***	36.34±7.25***	36.79±5.32***
4	100±0.01	68.09±12.48*	67.67±14.1*	46.07±6.04***	36.44±3.26***
5	100±0.03	75.22±13.59	97.13±20.25	62.43±9.13**	60.83±10.95**
6	100±0.0028	118.88±10.97	113.29±10.63	97.39±3.73	100.04±3.94

RAW 48hr without LPS (n=3)

RAW 48hr without LPS	0uM	10uM	20uM	50uM	100uM
1	100±1.45	102.68±4.76	108.56±3.57**	142.88±7.9	175.31±2.22***
2	100±1.45	92.78±1.89*	107.38±1.67*	122.57±5.2*	141.24±5.04**
3	100±0.58	99.96±2.23	93.72±0.49***	97.58±0.48**	108.68±2.49*
4	100±1.15	93.71±5.11	89.69±4.35	94.17±4.42	97.63±3.89
5	100±2	91.63±1.92*	93.39±2.62	95.73±9.02	107.62±5.6
6	100±1.76	104.01±15.64	96.84±2.38	99.75±5.03	102.26±3.07
