# Supplementary material for: Anti-inflammatory activity of electron-deficient organometallics
Source: R Soc Open Sci. 2017 Nov 29;4(11):170786. doi: 10.1098/rsos.170786 (PMC5717645; doi:10.1098/rsos.170786)
Supplement: Figures [file rsos170786supp3.pdf]

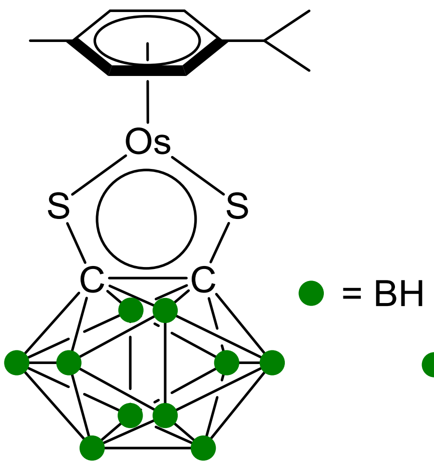

1

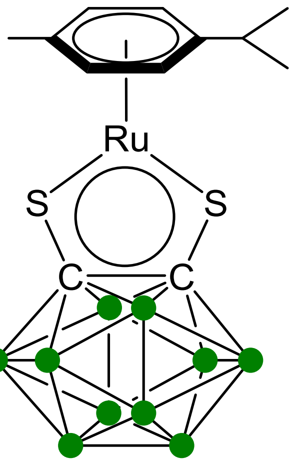

2

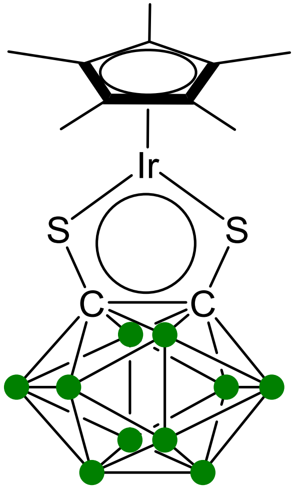

3

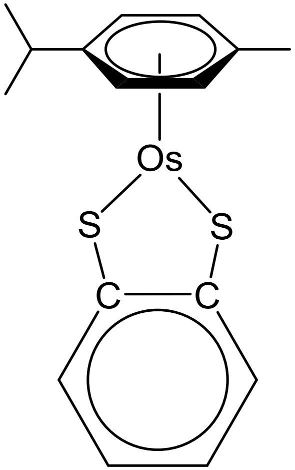

4

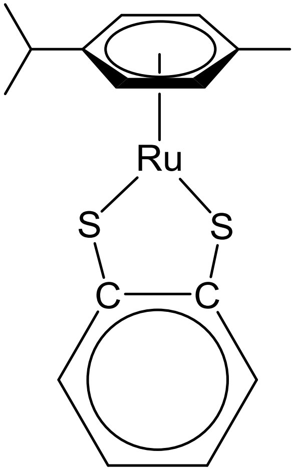

5

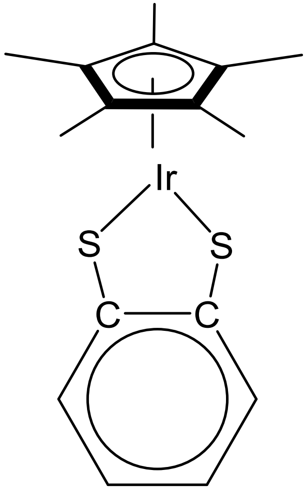

6

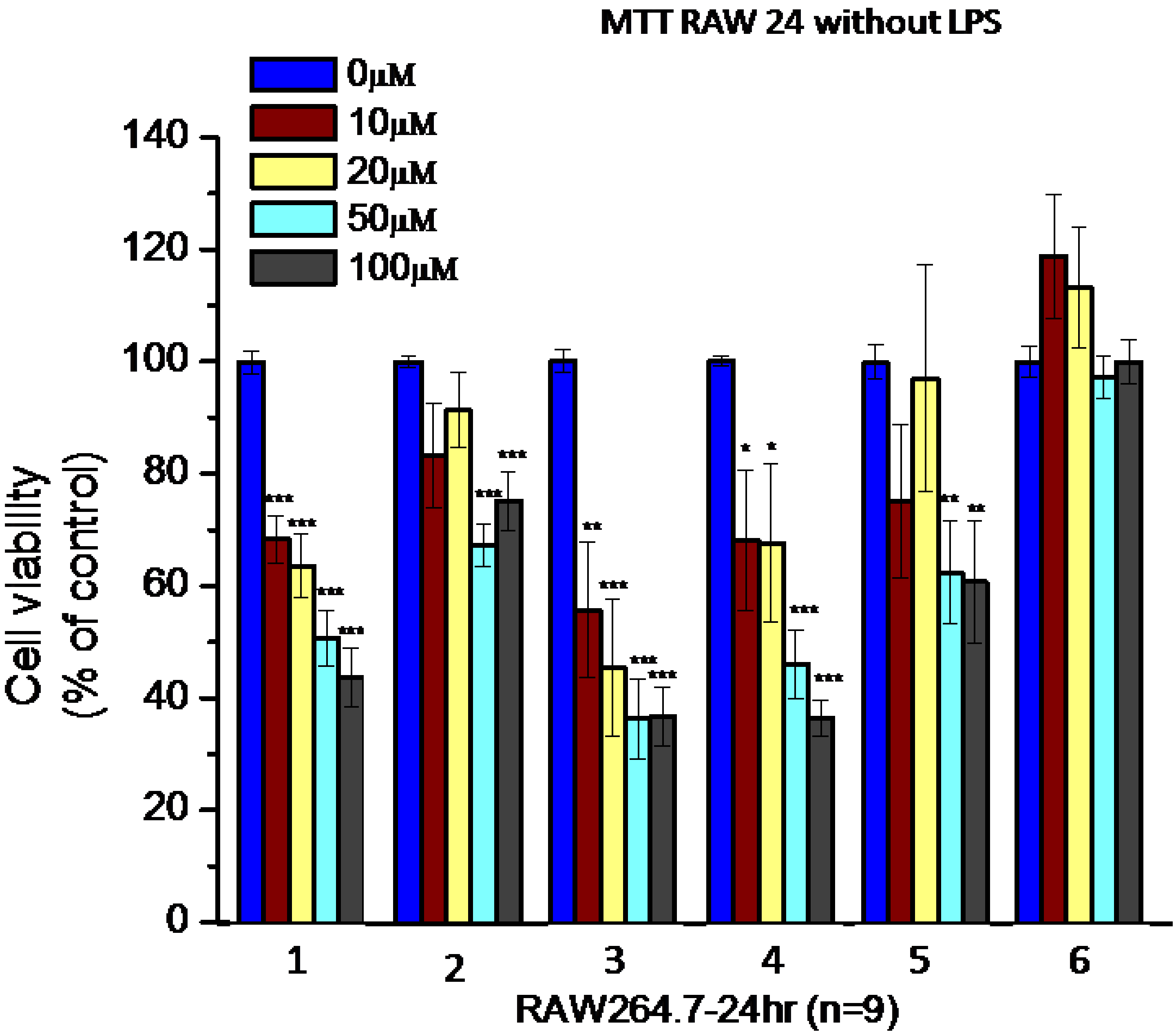
MTT RAW 24 without LPS
0μM
10μM
20μM
50μM
100μM
140
120
100
80
60
40
20
0
Cell viability
(% of control)
1
2
3
4
5
6
RAW264.7-24hr (n=9)

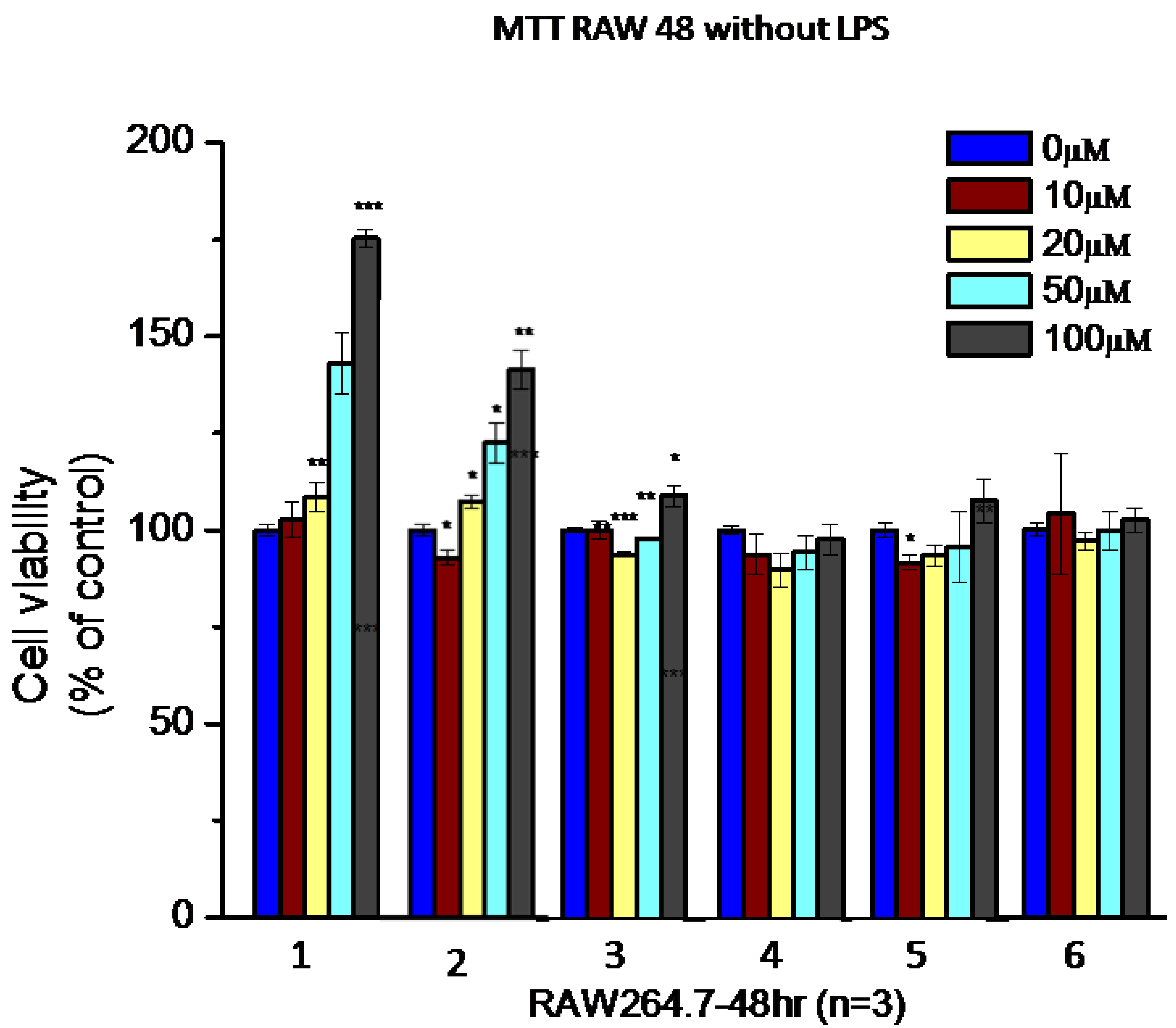

MTT RAW 48 without LPS
Cell viability
(% of control)
200
150
100
50
0
0μM
10μM
20μM
50μM
100μM
1
2
3
4
5
6
RAW264.7-48hr (n=3)

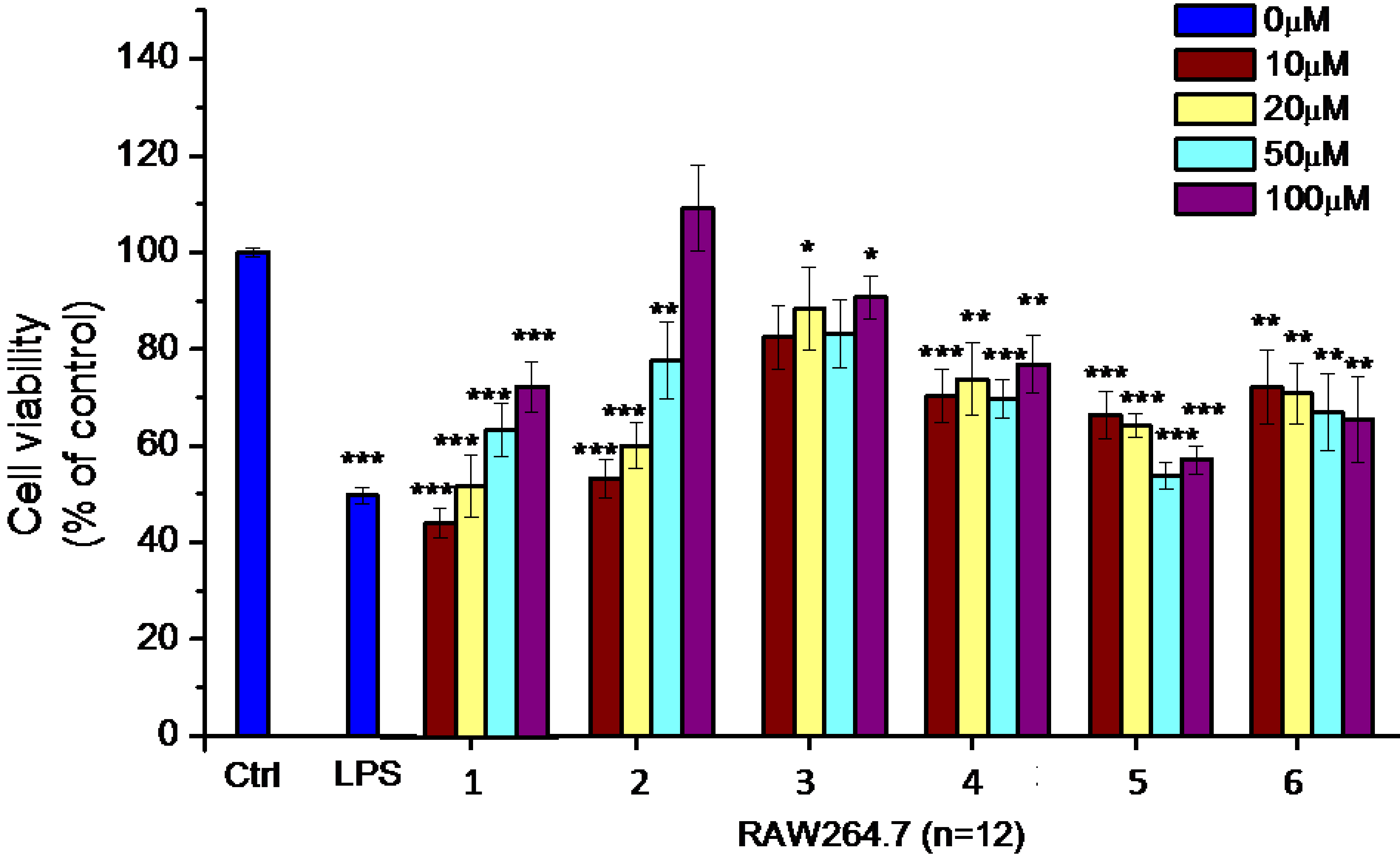

0μM
10μM
20μM
50μM
100μM
Cell viability
(% of control)
140
120
100
80
60
40
20
0
Ctrl
LPS
1
2
3
4
5
6
RAW264.7 (n=12)

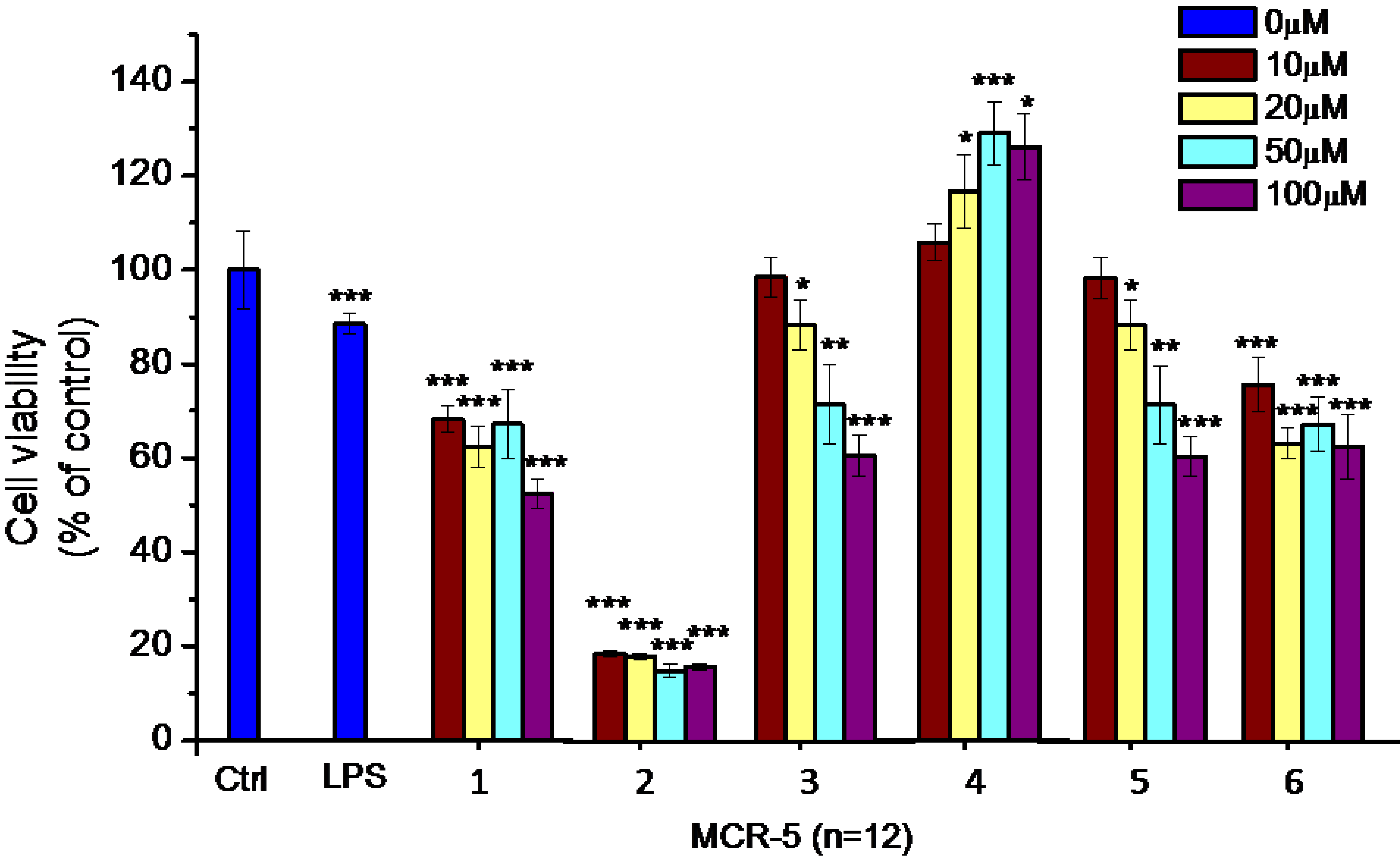

Cell viability
(% of control)
0
20
40
60
80
100
120
140
0μM
10μM
20μM
50μM
100μM
Ctrl
LPS
1
2
3
4
5
6
MCR-5 (n=12)

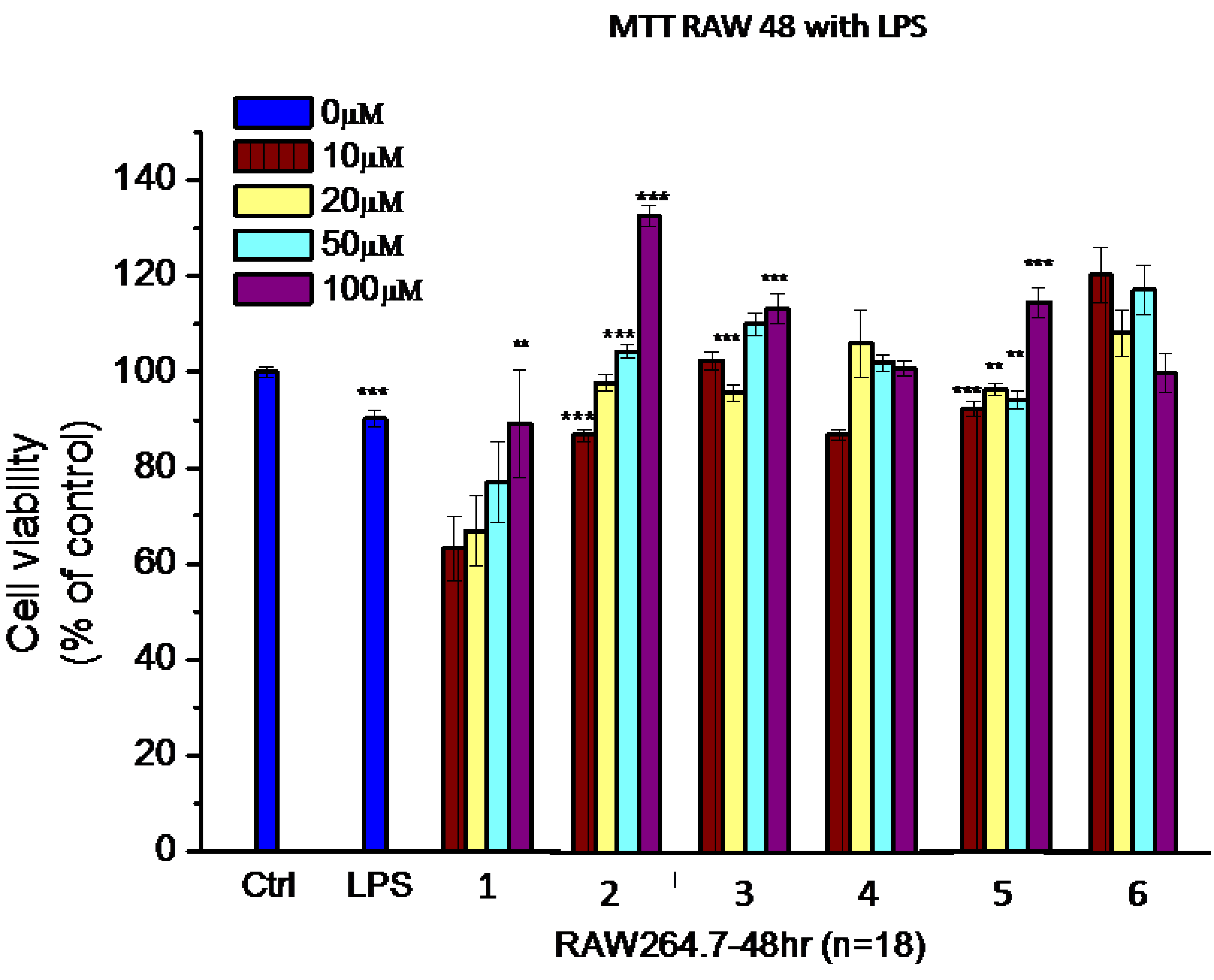

MTT RAW 48 with LPS
0μM
10μM
20μM
50μM
100μM
Cell viability
(% of control)
0
20
40
60
80
100
120
140
***
**
***
***
***
***
***
***
***
**
**
***
Ctrl
LPS
1
2
3
4
5
6
RAW264.7-48hr (n=18)

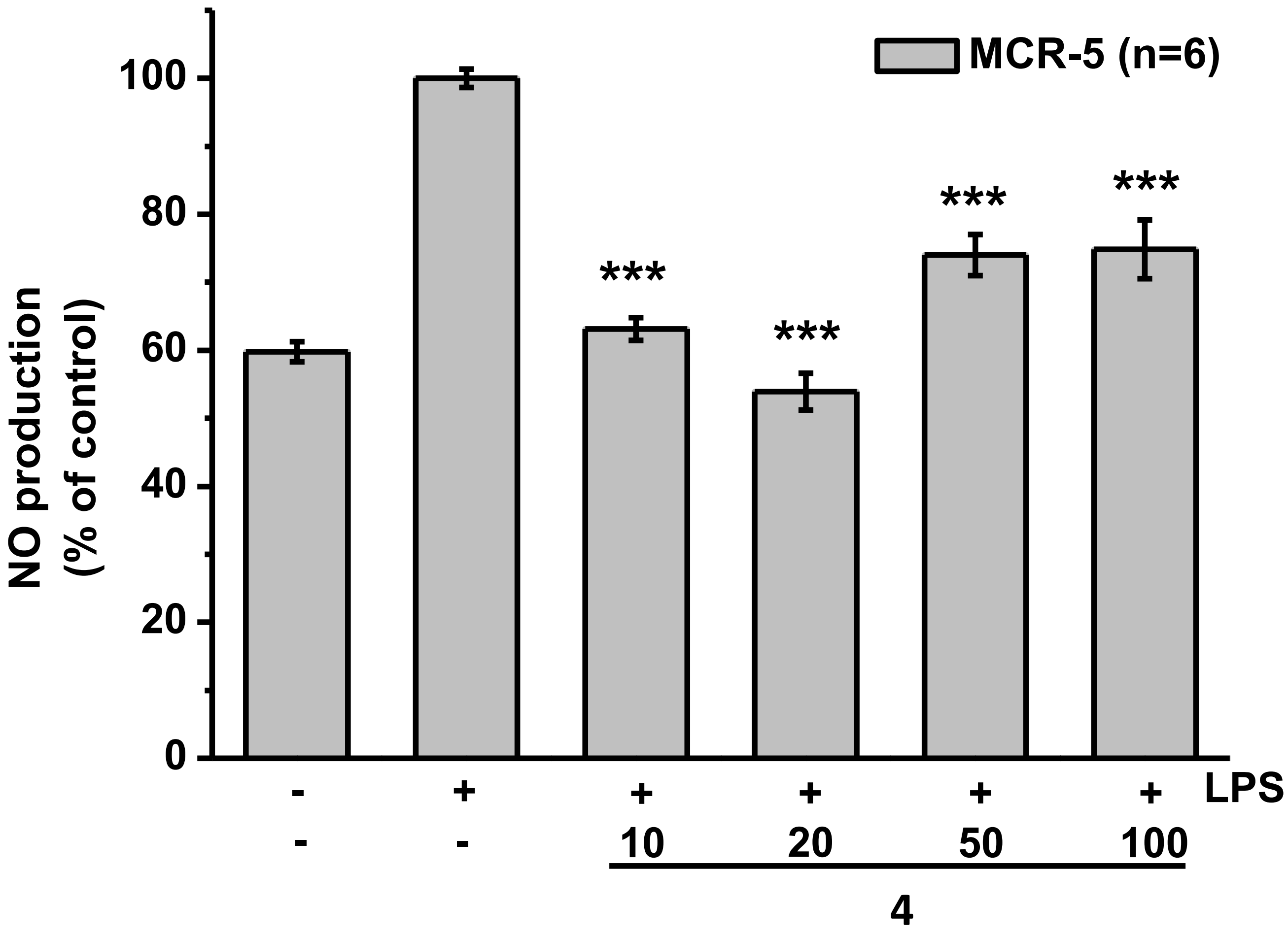
MCR-5 (n=6)
NO production
(% of control)
0
20
40
60
80
100
***
***
***
***
-
+
+
+
+
+
LPS
-
-
10
20
50
100
4

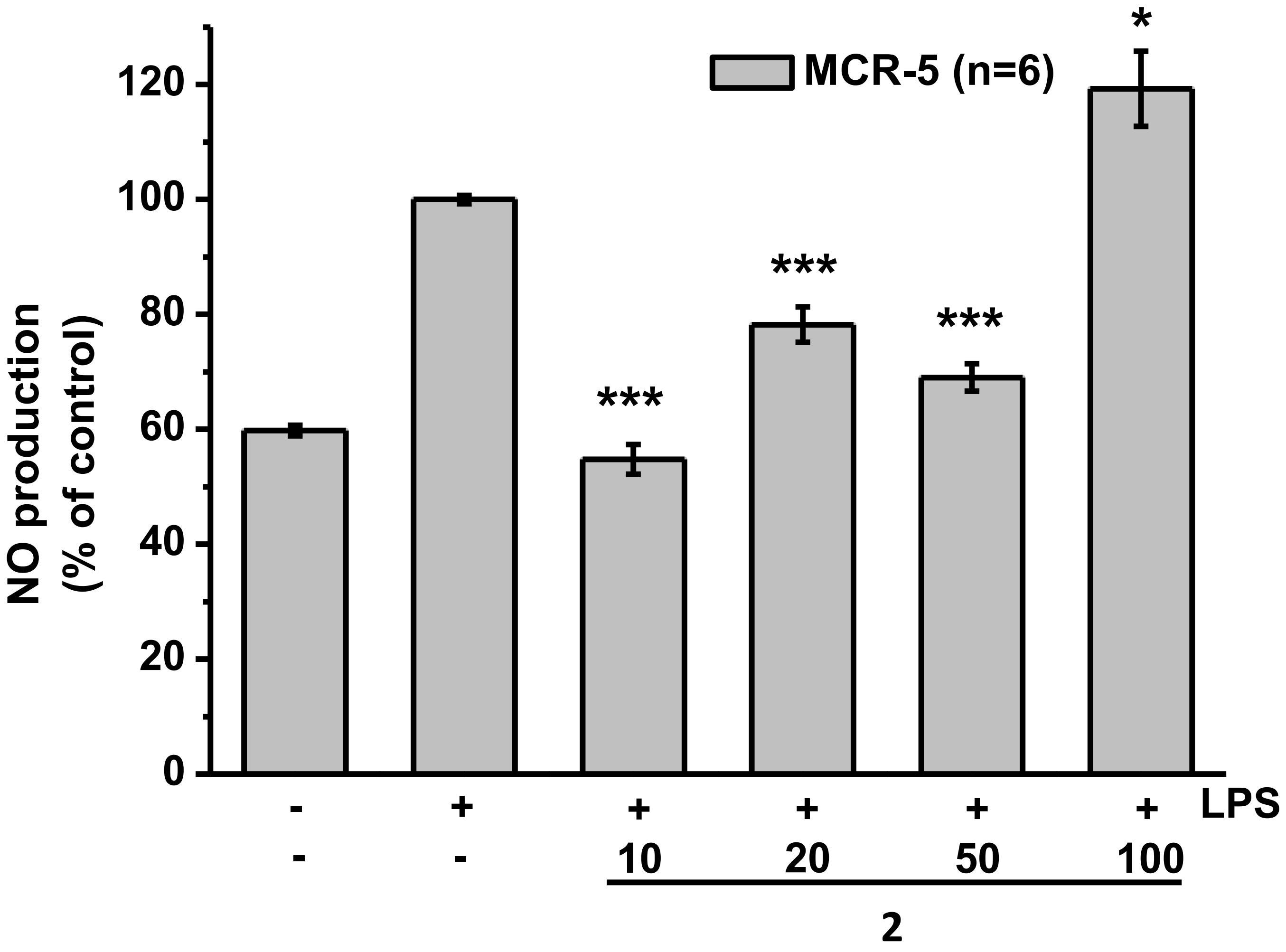
MCR-5 (n=6)
NO production
(% of control)
0
20
40
60
80
100
120
***
***
***
*
- + + + + + LPS
- - 10 20 50 100
2

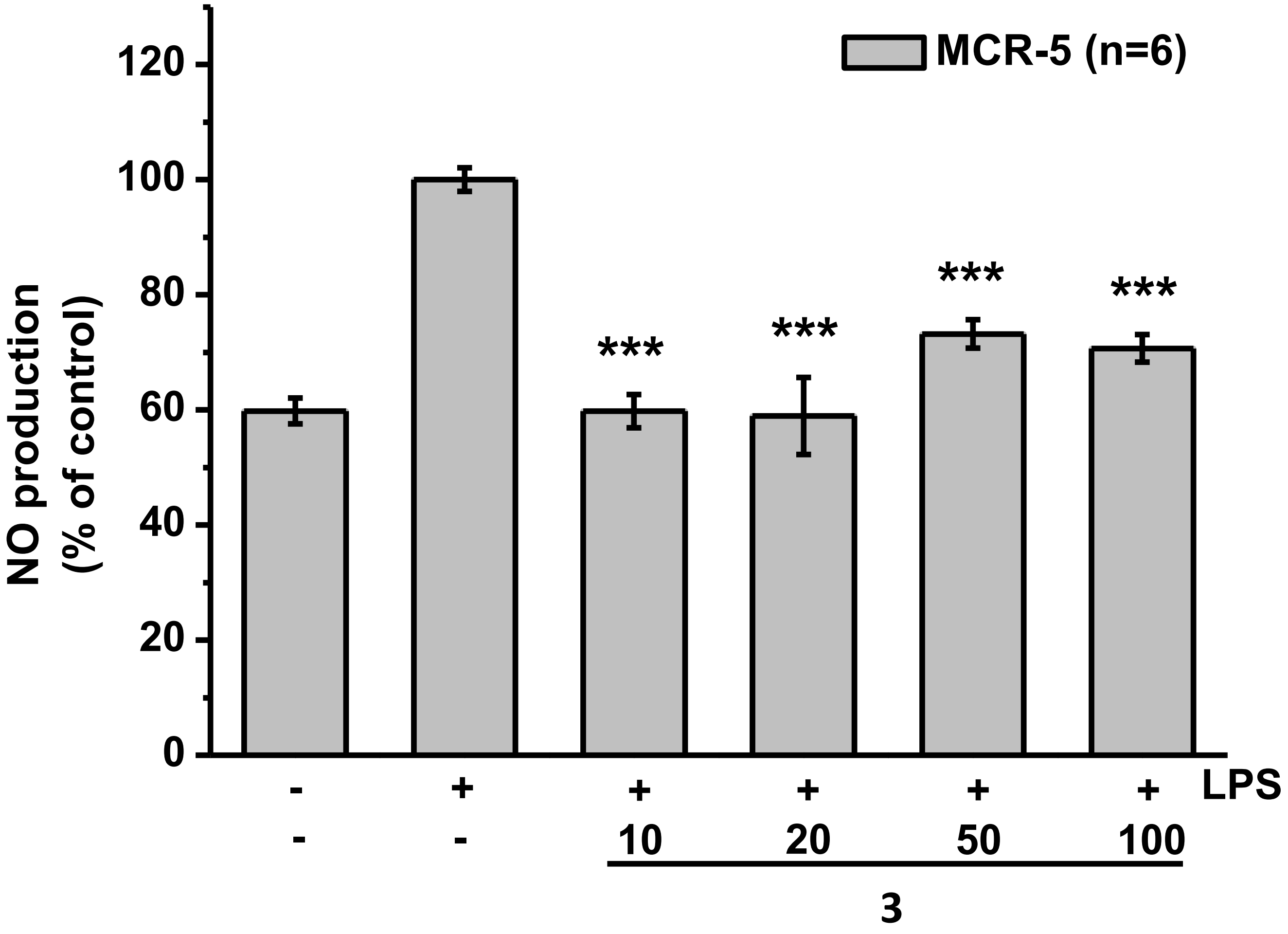

MCR-5 (n=6)
NO production
(% of control)
0
20
40
60
80
100
120
***
***
***
***
-
+
+
+
+
+
LPS
-
-
10
20
50
100
3

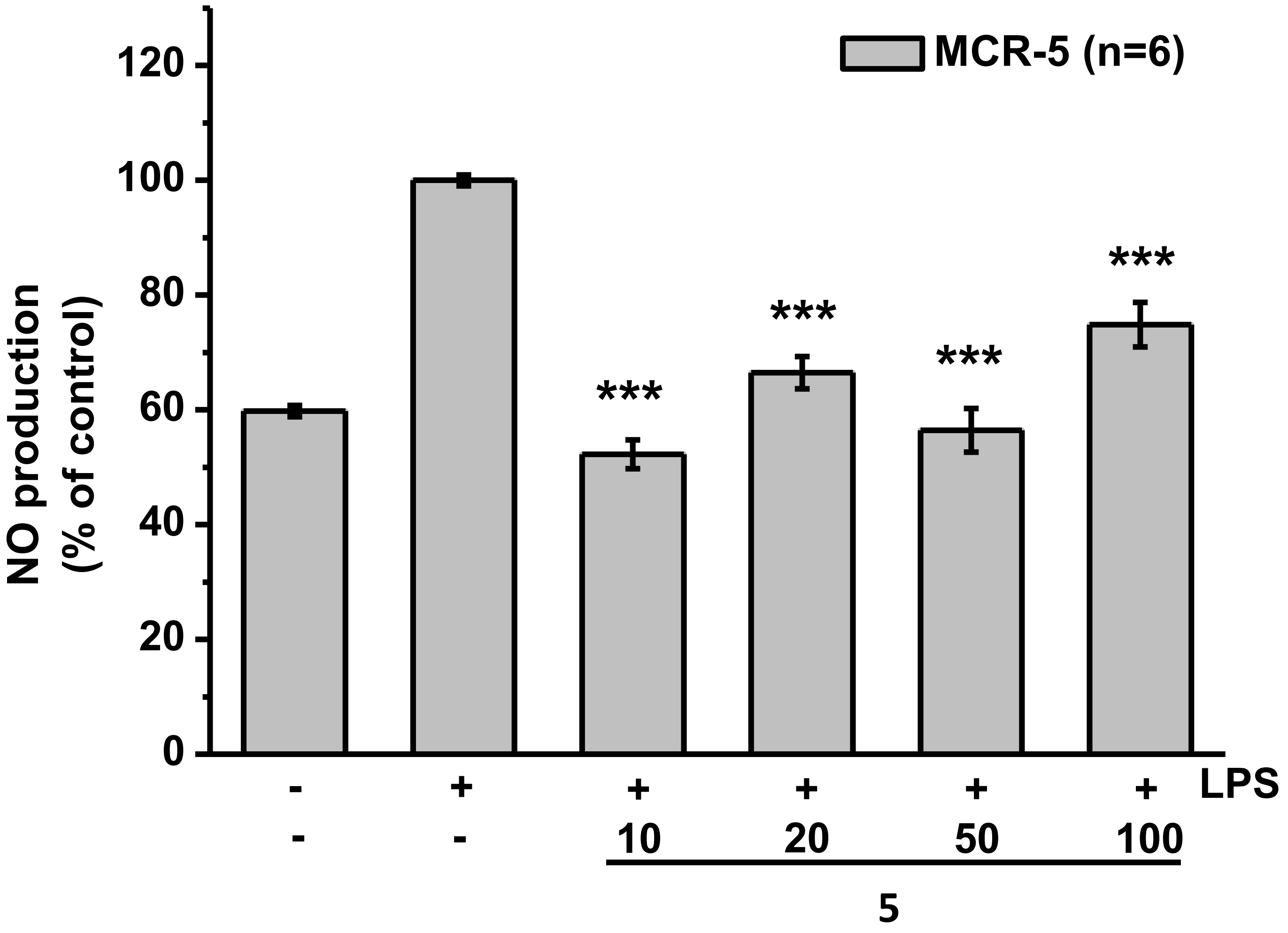

MCR-5 (n=6)
NO production
(% of control)
0
20
40
60
80
100
120
***
***
***
***
- + + + + + LPS
- - 10 20 50 100
5

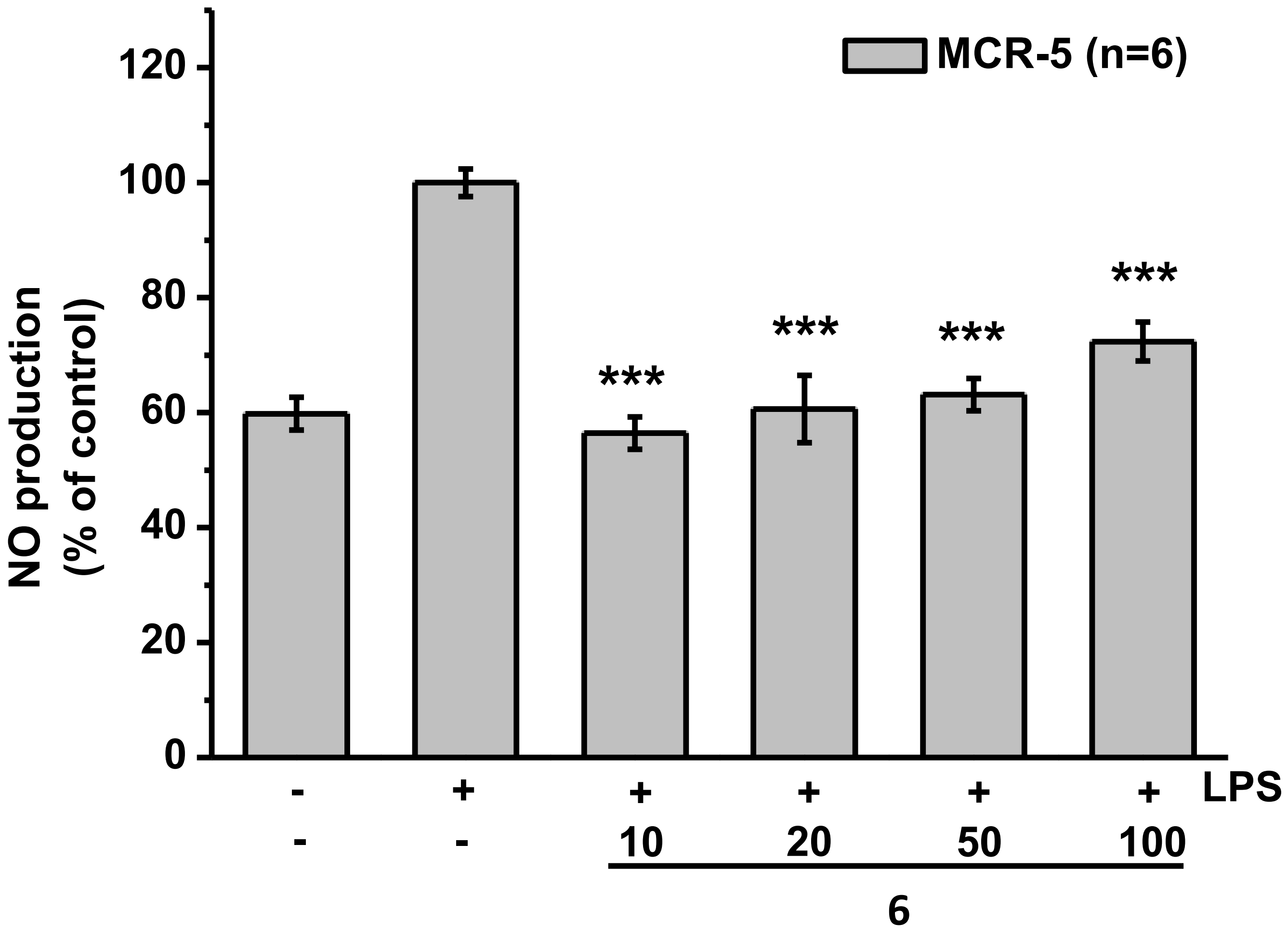
MCR-5 (n=6)
NO production
(% of control)
0
20
40
60
80
100
120
***
***
***
***
- + + + + + LPS
- - 10 20 50 100
6

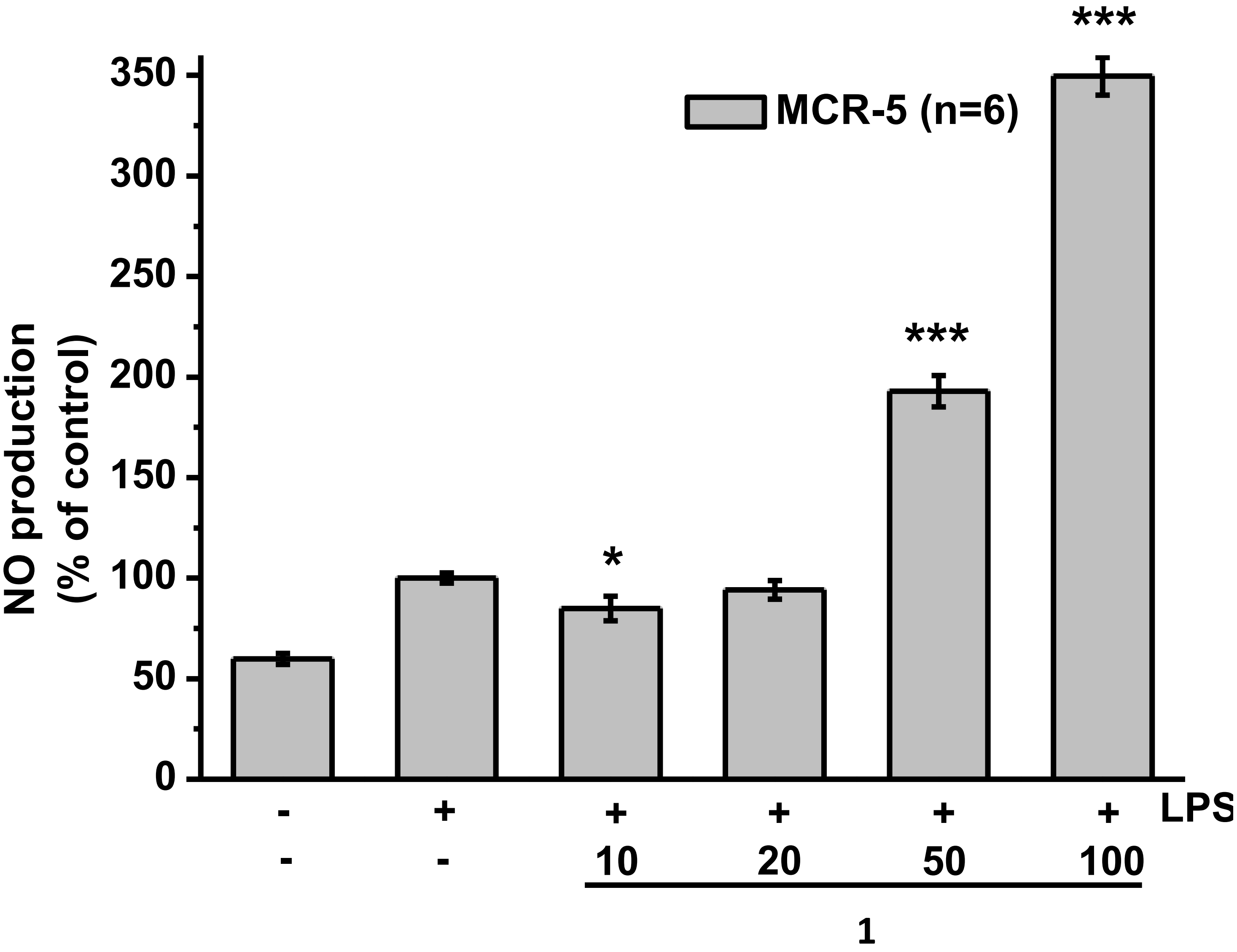

MCR-5 (n=6)
NO production
(% of control)
0
50
100
150
200
250
300
350
*
***
***
LPS
-
+
+
+
+
+
-
-
10
20
50
100
1

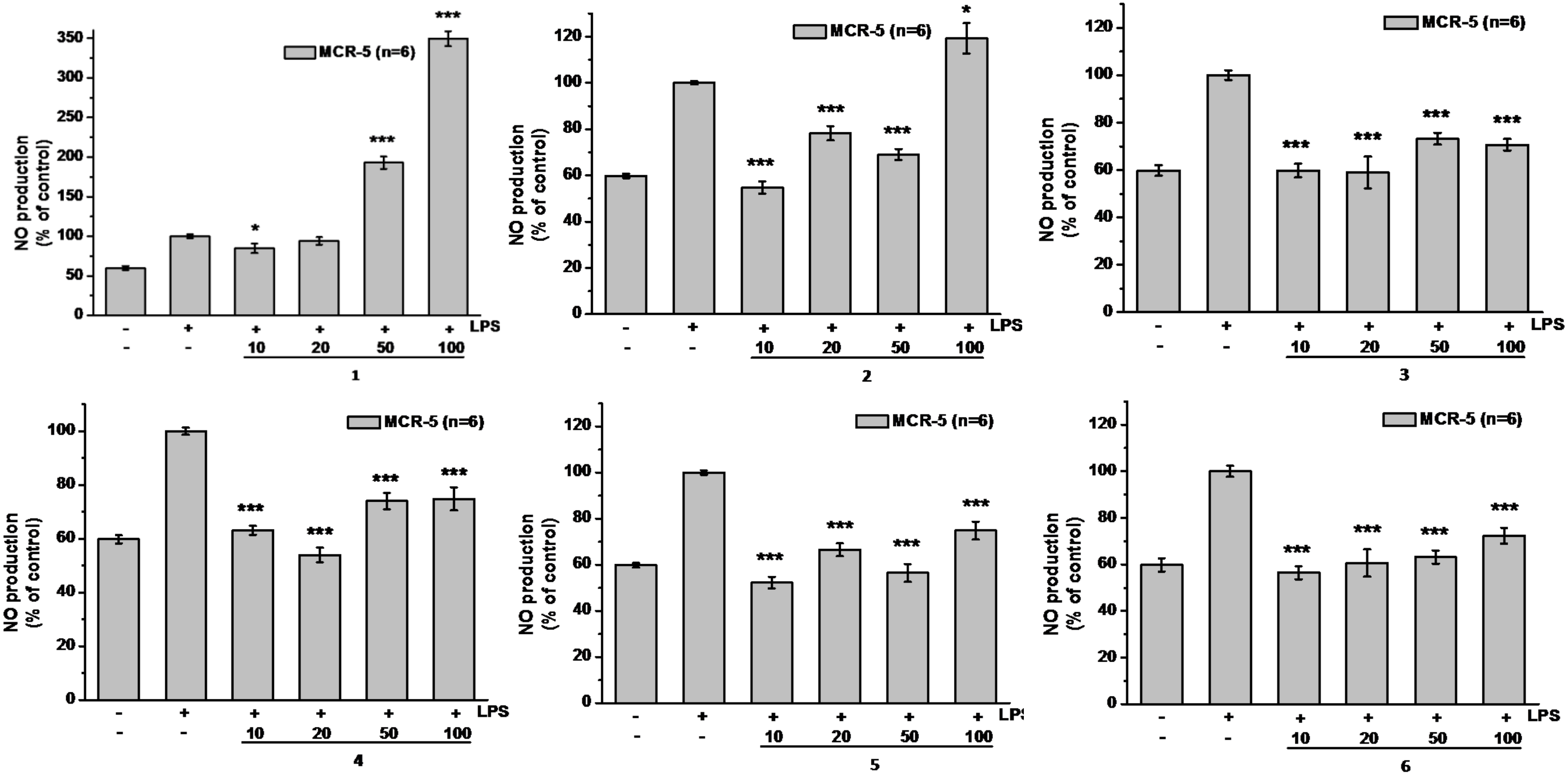

MCR-5 (n=6)
NO production
(% of control)
0
50
100
150
200
250
300
350
*
***
***
LPS
10
20
50
100
1
2
3
4
5
6
0
20
40
60
80
100
120

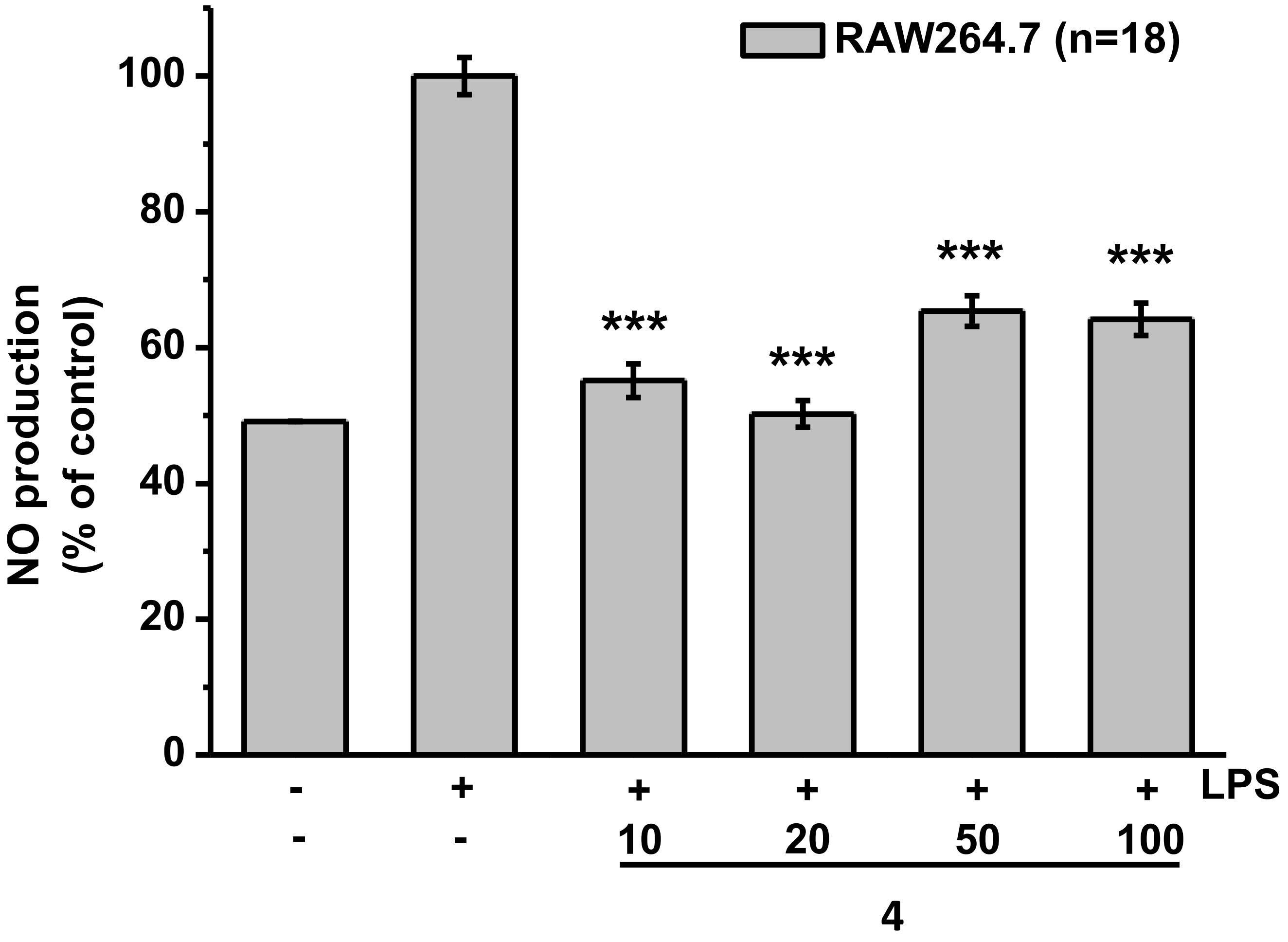
RAW264.7 (n=18)
NO production
(% of control)
0
20
40
60
80
100
***
***
***
***
LPS
- + + + + +
- - 10 20 50 100
4

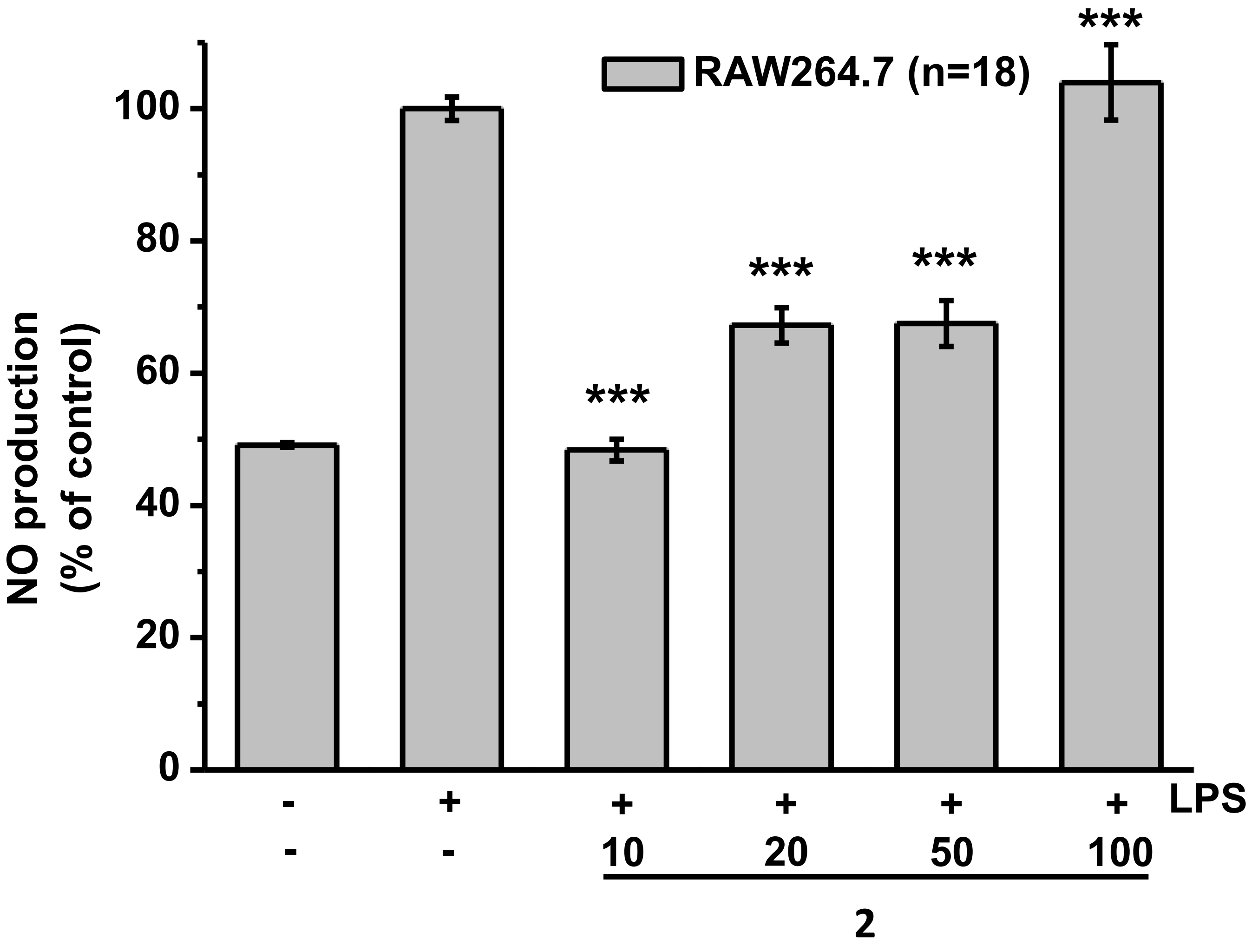

RAW264.7 (n=18)
NO production
(% of control)
0
20
40
60
80
100
***
***
***
***
-
+
+
+
+
+
LPS
-
-
10
20
50
100
2

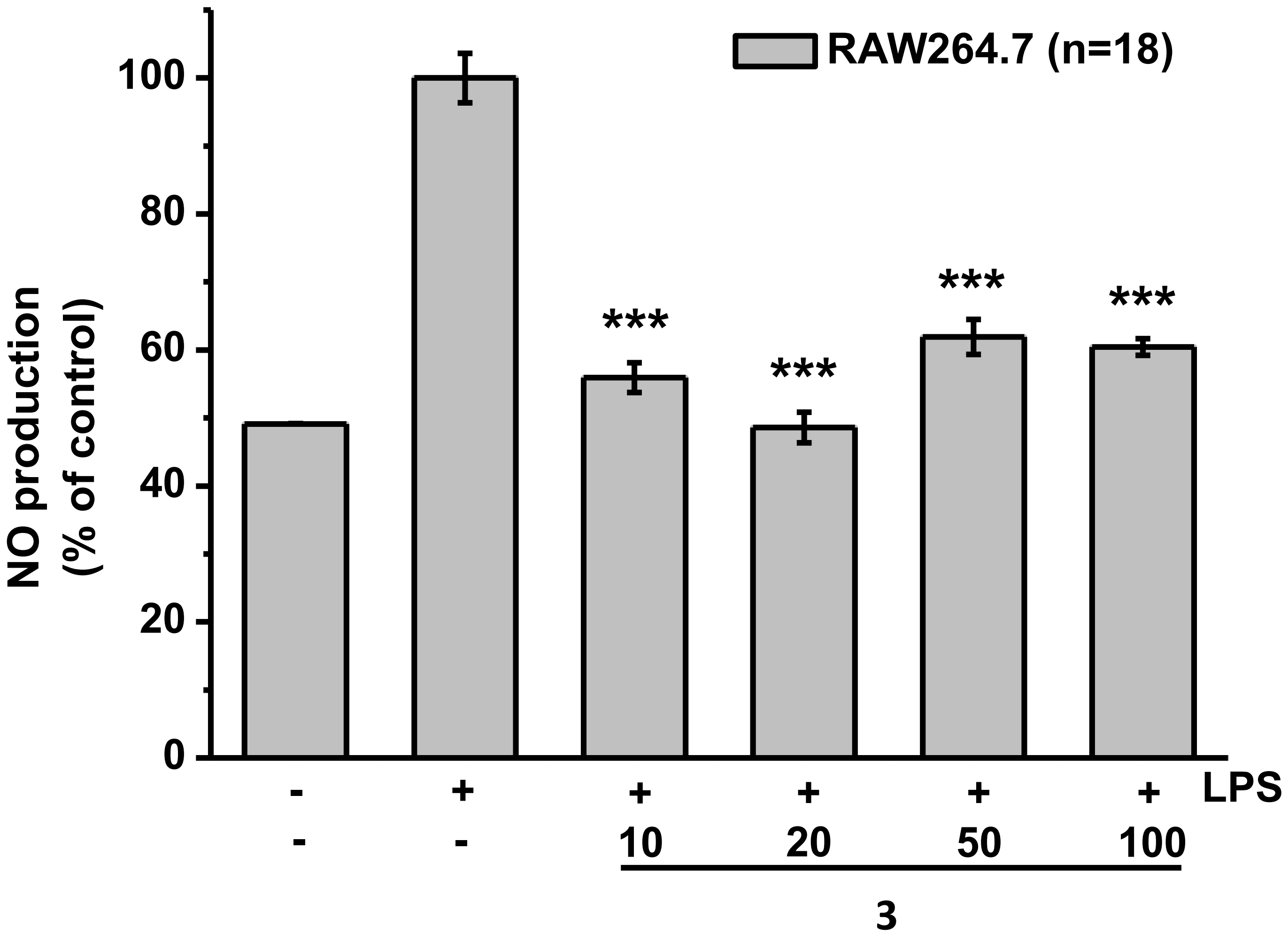

RAW264.7 (n=18)
NO production
(% of control)
0
20
40
60
80
100
***
***
***
***
- + + + + + LPS
- - 10 20 50 100
3

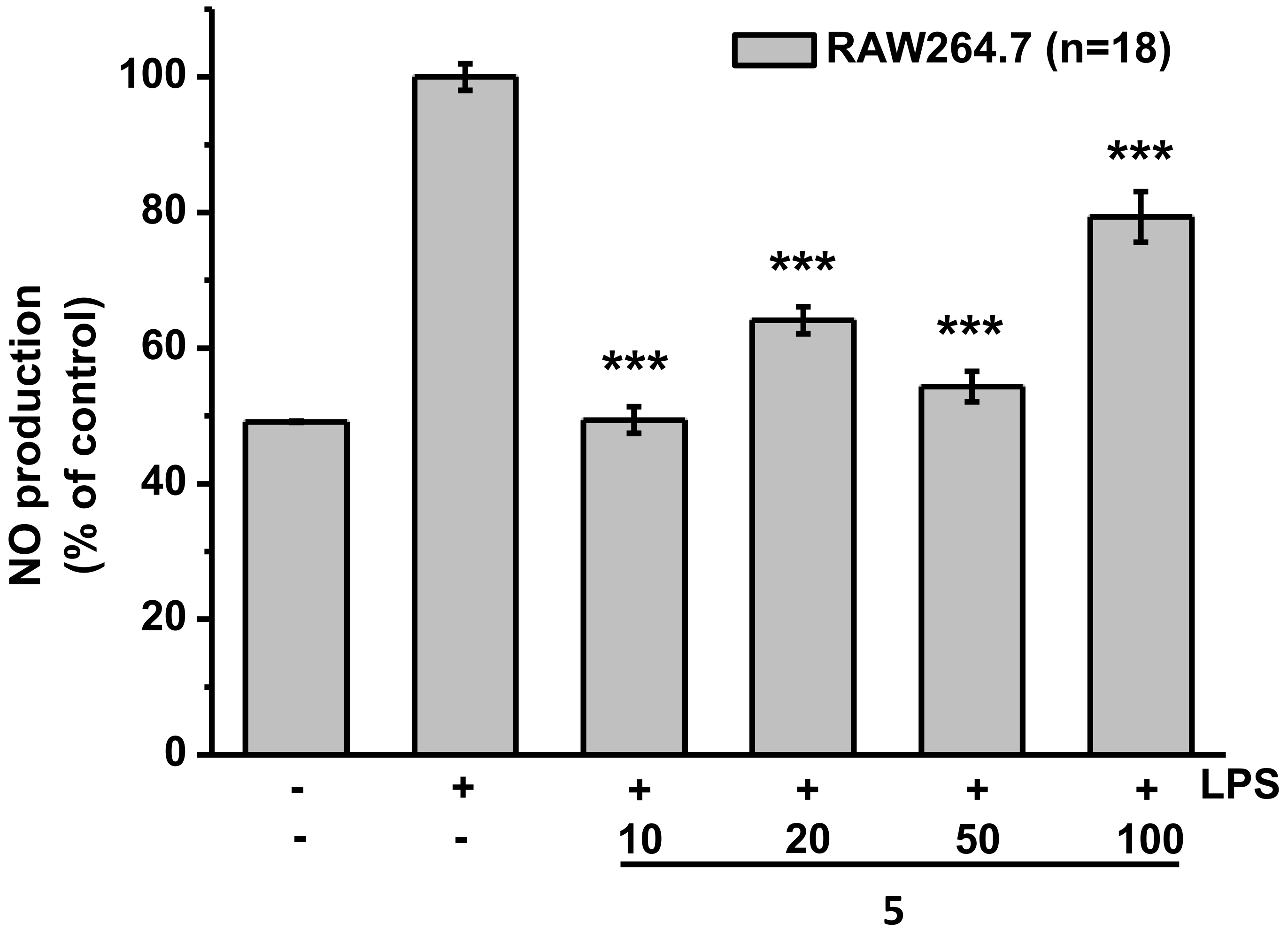
RAW264.7 (n=18)
NO production
(% of control)
0
20
40
60
80
100
***
***
***
***
-
+
+
+
+
+
LPS
-
-
10
20
50
100
5

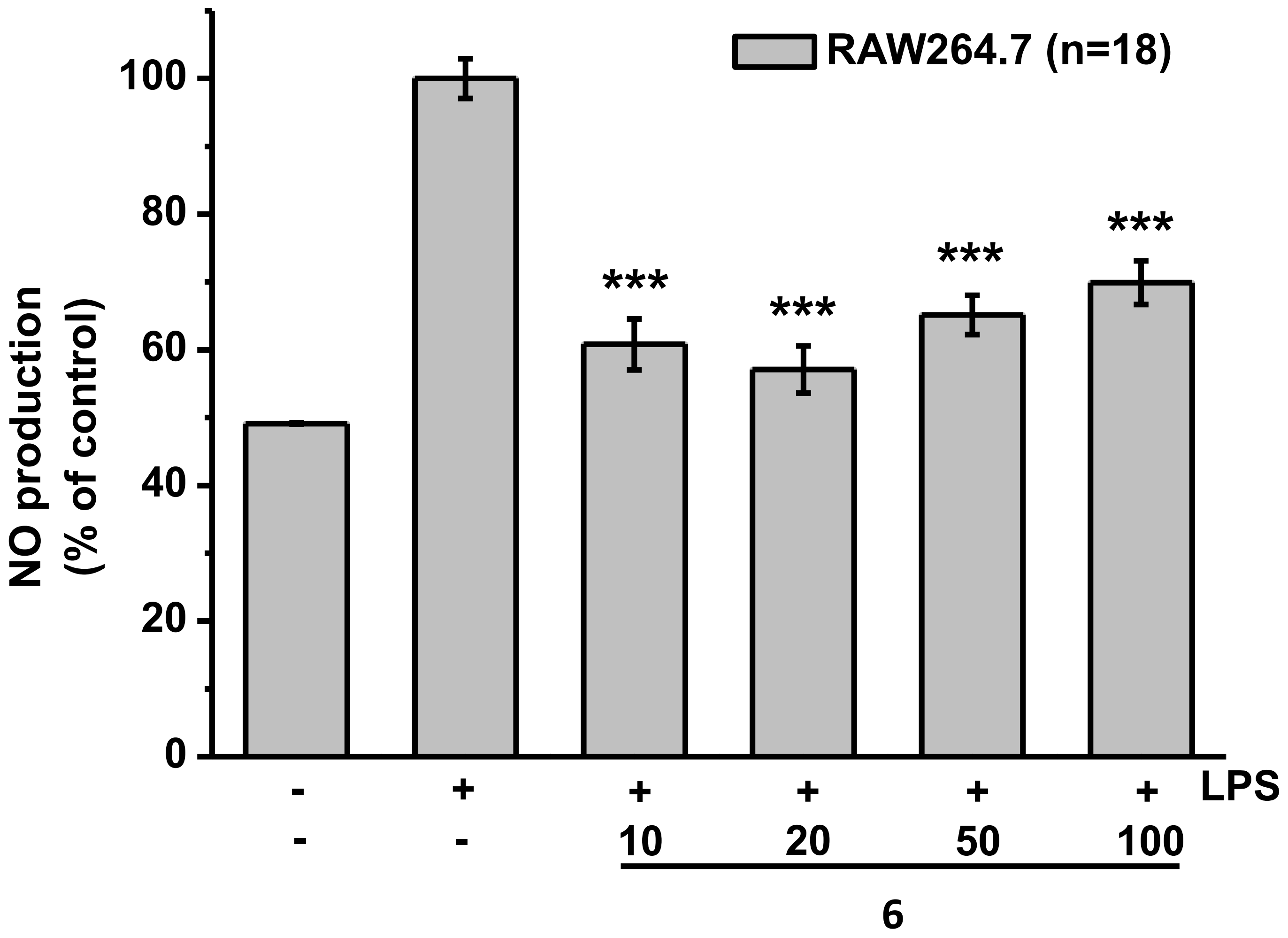
RAW264.7 (n=18)
NO production
(% of control)
0
20
40
60
80
100
***
***
***
***
- + + + + + LPS
- - 10 20 50 100
6

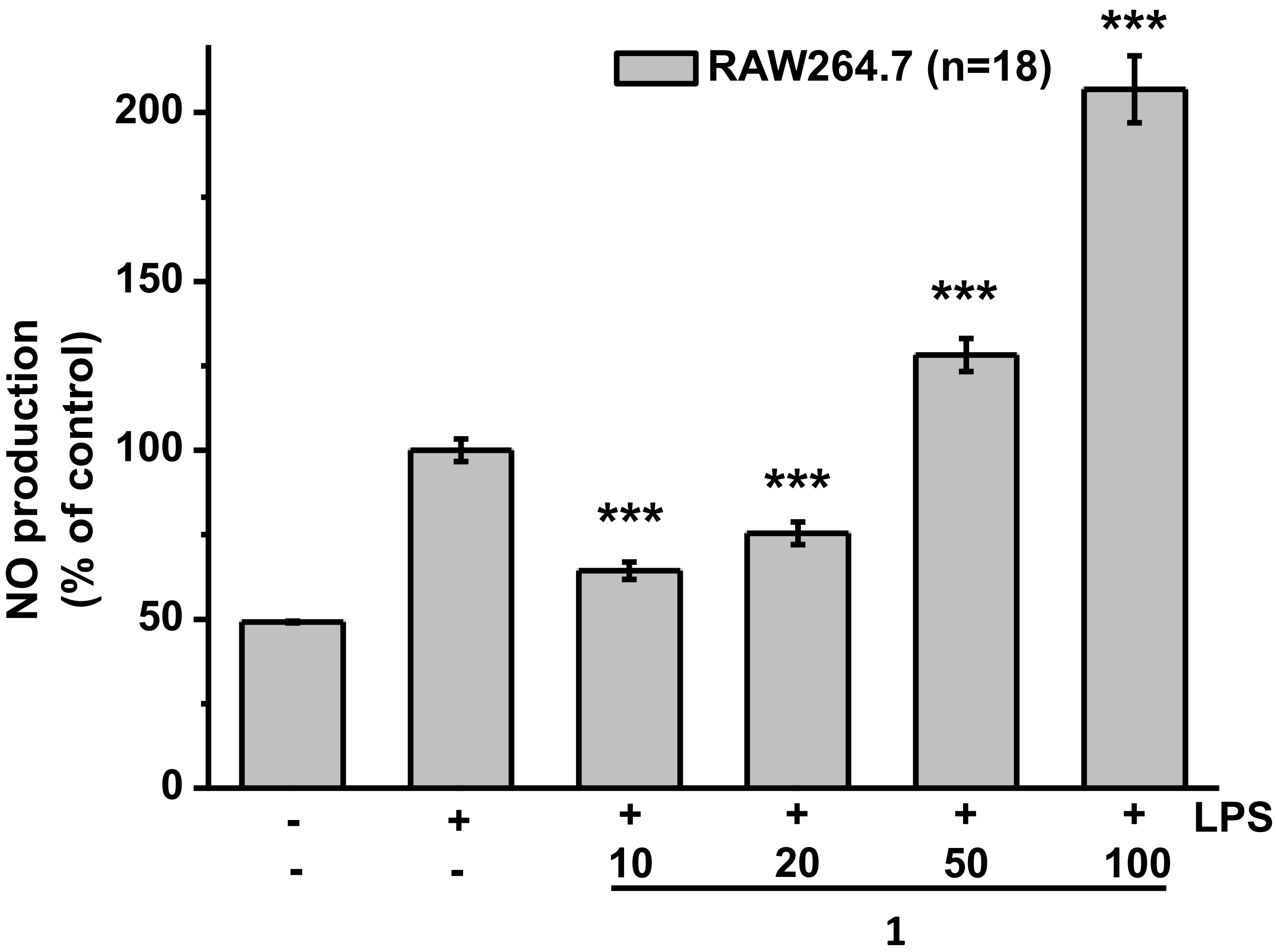
RAW264.7 (n=18)
NO production
(% of control)
0
50
100
150
200
***
***
***
***
-
+
+
+
+
+
LPS
-
-
10
20
50
100
1

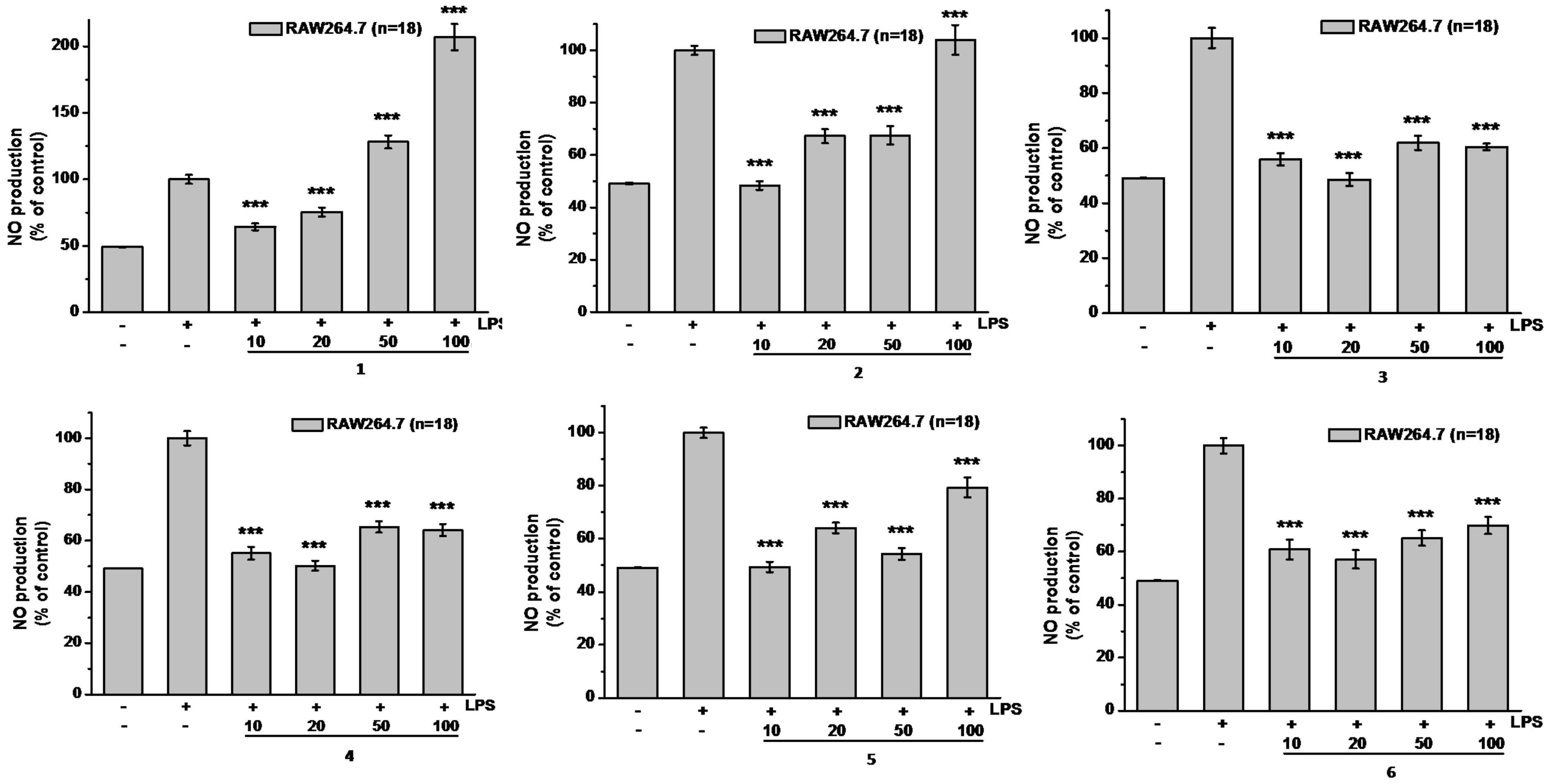
RAW264.7 (n=18)
NO production (% of control)
***
LPS
- + + + + +
- - 10 20 50 100
1
2
3
4
5
6

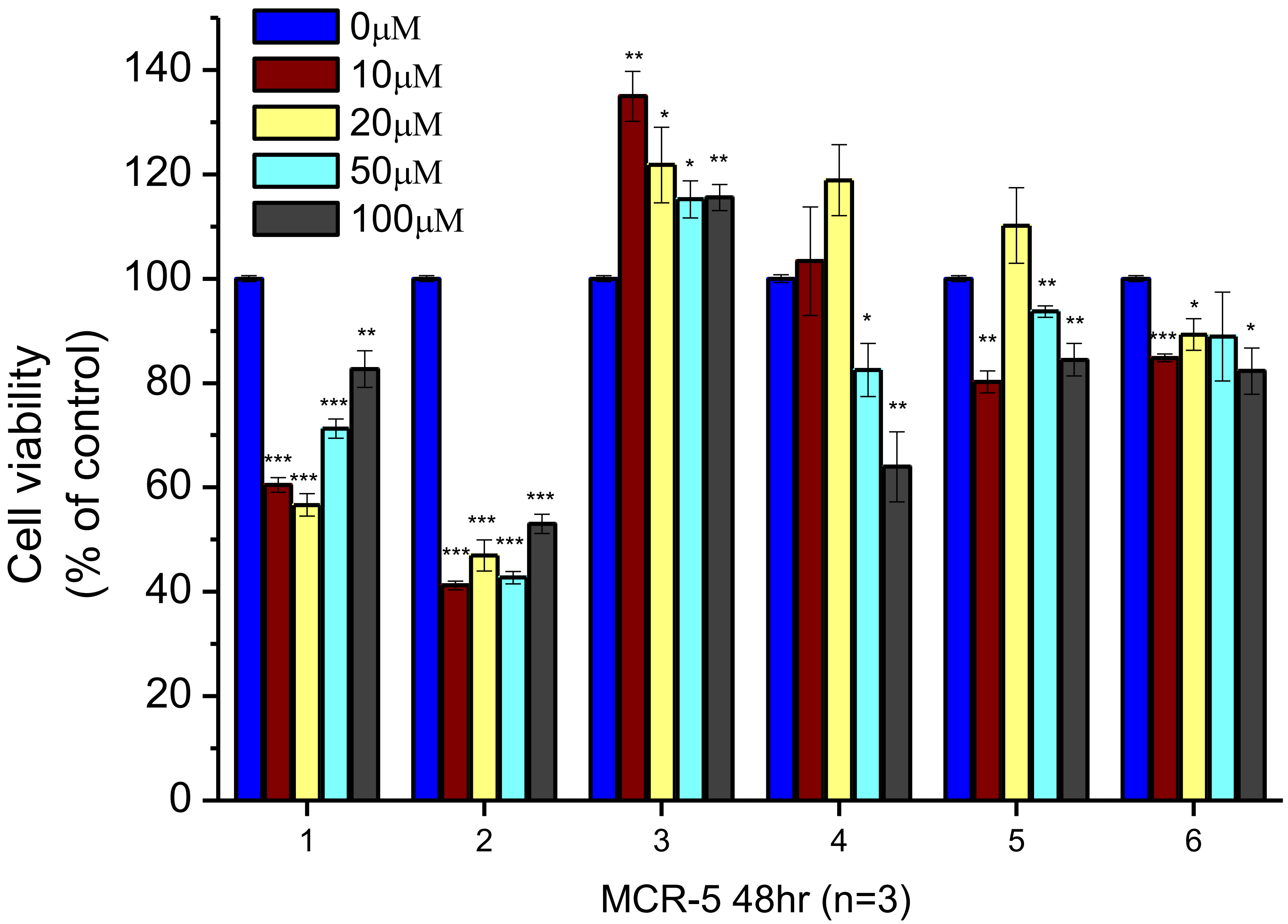

0μM
10μM
20μM
50μM
100μM
Cell viability
(% of control)
MCR-5 48hr (n=3)
0
20
40
60
80
100
120
140
1
2
3
4
5
6

MCR-5 48hr without LPS

MCR-5	CE32 4	JT020 2	CE40 3
0uM	100±0.76	100±0.3	100±0.29
10uM	103.39±10.4	41.25±0.84	134.96±4.81
20	118.85±6.8	46.96±2.99	121.79±7.27
50	82.49±5.06	42.73±1.18	115.22±3.54
100	63.97±6.7	53.01±1.82	115.55±2.49

MCR-5	CE44 5	CE47 6	CE49 1
0uM	100±0.23	100±0.58	’ 100±0.27
10uM	80.25±2.1	84.82±0.76	60.50±1.41
20	110.20±7.22	89.28±3.04	56.65±2.19
50	93.73±1.1	88.91±8.52	71.27±1.86
100	84.46±3.13	82.32±4.44	82.67±3.53

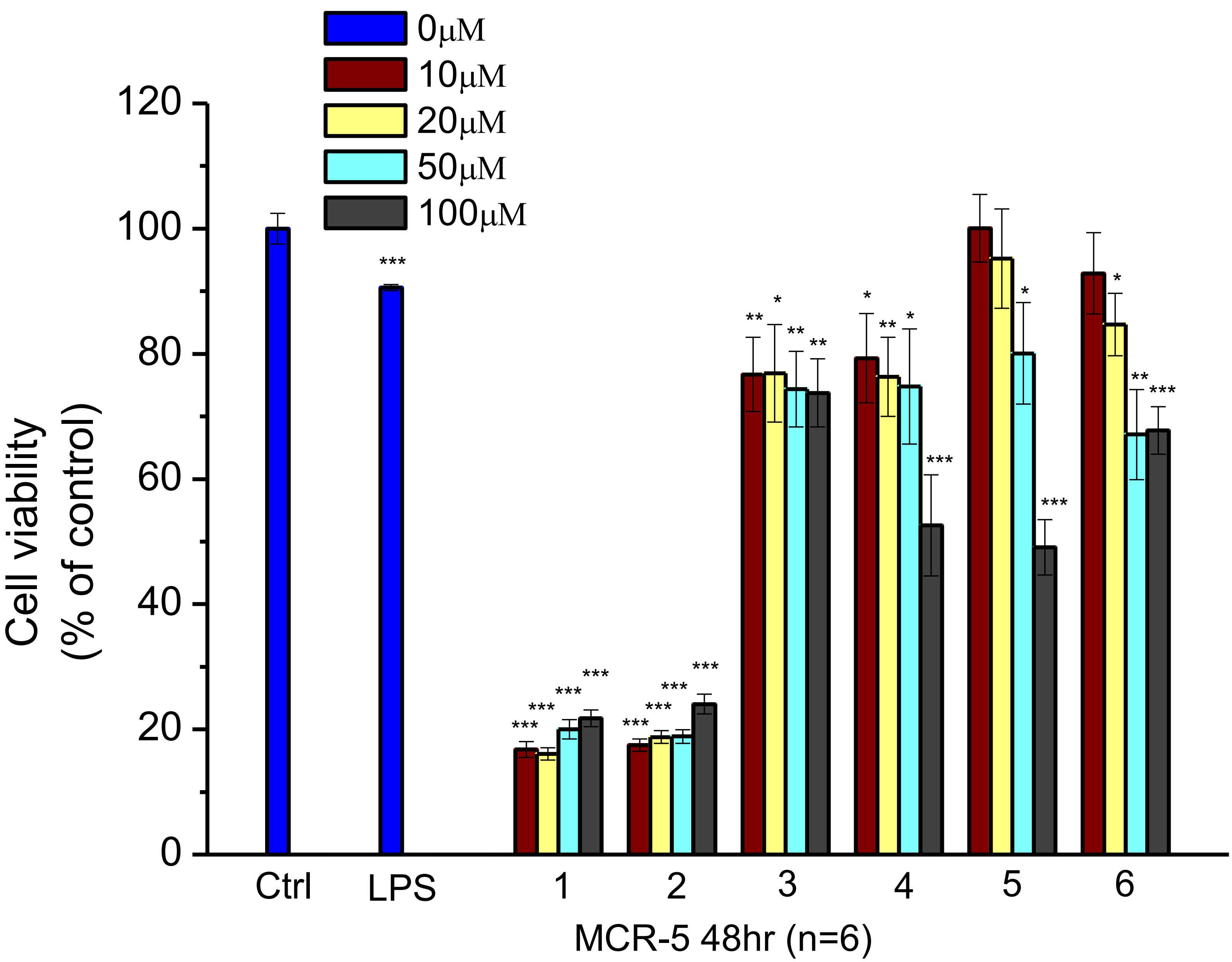
0μM
10μM
20μM
50μM
100μM
Cell viability
(% of control)
120
100
80
60
40
20
0
***
Ctrl
LPS
1
2
3
4
5
6
MCR-5 48hr (n=6)

MCR-5	
Ctrl	100±2.47
LPS	90.6±0.48

MCR-5 48 hr with LPS

[illegible]CR-5	CE32 4	JT020 2	CE40 3
10uM	79. 30±7. 14	17. 49±1. 01	76. 69±5. 94
20	76. 30±6. 33	18. 78±0. 99	76. 89±7. 81
50	74. 78±9. 19	18. 86±1. 07	74. 37±6. 06
100	52. 59±8. 08	24. 04±1. 6	73. 76±5. 43

MCR-5	CE44 5	CE47 6	CE49 1
10uM	100. 07±5. 41	92. 85±6. 49	16. 77±1. 27
20	95. 21±7. 93	84. 68±5. 01	16. 07±0. 97
50	80. 08±8. 09	67. 10±7. 18	19. 99±1. 56
100	49. 05±4. 42	67. 77±3. 79	21. 77±1. 33

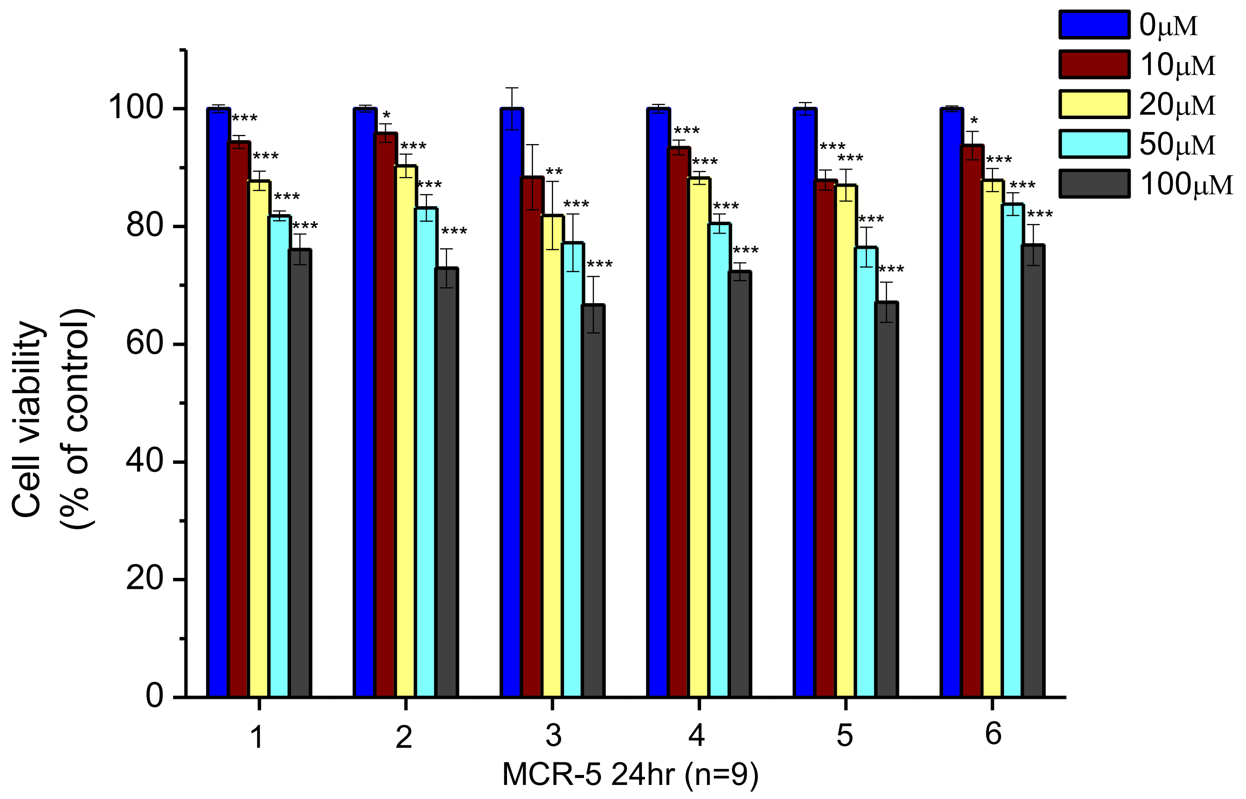
0μM
10μM
20μM
50μM
100μM
Cell viability
(% of control)
MCR-5 24hr (n=9)

MCR -5 24hr	0uM	10uM	20uM	50uM	100uM
1	100±0.67	94.35±1.1***	87.76±1.65***	81.8±0.85***	76.11±2.61***
2	100±0.58	95.87±1.57*	90.31±1.99***	83.16±2.25***	72.9±3.34***
3	100±3.56	88.36±5.54	81.88±5.81**	77.26±4.89***	66.7±4.78***
4	100±0.76	93.4±1.25***	88.26±1.1***	80.49±1.63***	72.33±1.5***
5	100±1.04	87.88±1.71***	87.03±2.68***	76.48±3.37***	67.12±3.41***
6	100±0.49	93.75±2.41*	87.89±1.97***	83.81±1.94***	76.87±3.48***
